# The NSD2/WHSC1/MMSET methyltransferase prevents cellular senescence‐associated epigenomic remodeling

**DOI:** 10.1111/acel.13173

**Published:** 2020-06-22

**Authors:** Hiroshi Tanaka, Tomoka Igata, Kan Etoh, Tomoaki Koga, Shin‐ichiro Takebayashi, Mitsuyoshi Nakao

**Affiliations:** ^1^ Department of Medical Cell Biology Institute of Molecular Embryology and Genetics Kumamoto University Kumamoto Japan; ^2^ Japan Agency for Medical Research and Development Tokyo Japan; ^3^ Present address: Sanford Burnham Prebys Medical Discovery Institute La Jolla CA USA; ^4^ Present address: Laboratory of Molecular & Cellular Biology Graduate School of Bioresources Mie University Tsu Japan

**Keywords:** cell cycle control, H3K36 methylation, NSD2/WHSC1/MMSET, retinoblastoma, senescence‐associated epigenomic remodeling, senescence‐associated metabolic remodeling

## Abstract

Senescent cells may possess the intrinsic programs of metabolic and epigenomic remodeling, but the molecular mechanism remains to be clarified. Using an RNAi‐based screen of chromatin regulators, we found that knockdown of the NSD2/WHSC1/MMSET methyltransferase induced cellular senescence that augmented mitochondrial mass and oxidative phosphorylation in primary human fibroblasts. Transcriptome analysis showed that loss of NSD2 downregulated the expression of cell cycle‐related genes in a retinoblastoma protein (RB)‐mediated manner. Chromatin immunoprecipitation analyses further revealed that NSD2 was enriched at the gene bodies of actively transcribed genes, including cell cycle‐related genes, and that loss of NSD2 decreased the levels of histone H3 lysine 36 trimethylation (H3K36me3) at these gene loci. Consistent with these findings, oncogene‐induced or replicative senescent cells showed reduced NSD2 expression together with lower H3K36me3 levels at NSD2‐enriched genes. In addition, we found that *NSD2* gene was upregulated by serum stimulation and required for the induction of cell cycle‐related genes. Indeed, in both mouse and human tissues and human cancer cell lines, the expression levels of *NSD2* were positively correlated with those of cell cycle‐related genes. These data reveal that NSD2 plays a pivotal role in epigenomic maintenance and cell cycle control to prevent cellular senescence.

## Graphical abstract

RNAi‐based screen revealed that loss of the NSD2 methyltransferase induces cellular senescence in human fibroblasts. Epigenomic and transcriptomic analyses revealed that NSD2 is enriched at transcribed gene bodies, including cell cycle‐promoting genes, and that loss of NSD2 decreases H3K36me3 levels at NSD2‐enriched gene loci. The expression of NSD2 is augmented by serum stimulation, which is required for induction of cell cycle‐promoting genes.

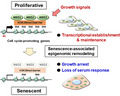

## 1 INTRODUCTION

Cellular senescence is induced by various stresses such as oncogene induction, telomere attrition, metabolic perturbation, and misregulation of chromatin. Senescent cells accumulate with age in a variety of tissues in mouse and human (Dimri et al., 1995; Jeyapalan & Sedivy, 2008) and cause tissue dysfunction at least in part through a proinflammatory program referred to as the senescence‐associated secretory phenotype (SASP) or senescence‐messaging secretome (Coppé et al., 2008; Kuilman & Peeper, 2009). Senescent cells often exhibit upregulated or downregulated metabolic activities possibly depending on inducers and time courses, including glycolysis, mitochondrial oxidative phosphorylation, and/or fatty acid oxidation, but the underlying mechanisms remain unclear (Hernandez‐Segura, Nehme, & Demaria, 2018; Pantazi et al., 2019; Takebayashi et al., 2015; Wiley & Campisi, 2016). Cellular metabolism likely plays a crucial role in regulating SASP because abnormal activation of mitochondria increases the expression of a subset of secretory proteins and promotes senescence (Correia‐Melo et al., 2016; Kaplon et al., 2013; Quijano et al., 2012). Moreover, dysfunction of mitochondria also leads to senescence, accompanied by a distinct secretory phenotype (Wiley et al., 2016). Thus, clarifying the effector mechanisms of senescence‐associated metabolic remodeling is important to understand the senescence program.

Chromatin regulators play an essential role in gene expression and metabolic regulation (Anan et al., 2018; Nakao, Anan, Araki, & Hino, 2019). Numerous epigenetic factors are implicated in the process of senescence and aging, such as DNA methylation, histone modification, and higher‐order chromatin structure. Thus, we hypothesized that (Booth & Brunet, 2016; Criscione, Teo, & Neretti, 2016; Sun, Yu, & Dang, 2018) certain chromatin regulators may be involved in the molecular basis of senescence‐associated metabolic and epigenomic remodeling. We previously reported that the SETD8 methyltransferase functions in senescence‐associated mitochondrial and ribosomal coactivation via histone H4 lysine 20 methylation (Tanaka et al., 2017).

NSD2, also known as WHSC1 or MMSET, is a methyltransferase that is responsible for mono‐, di‐, and/or tri‐methylation of histone H3 lysine 36 (H3K36me1, H3K36me2, and H3K36me3, respectively). NSD2 has also been closely associated with human diseases. As an oncogenic protein, NSD2 is overexpressed in a variety of cancer cells (Vougiouklakis, Hamamoto, Nakamura, & Saloura, 2015), and knockdown of NSD2 decreases proliferation in cancer cells at least in part by loss of H3K36me2 levels (García‐Carpizo et al., 2016; Kuo et al., 2011). In normal cell types, haploinsufficiency of *NSD2* causes developmental growth delay, the so‐called Wolf‐Hirschhorn syndrome (Boczek et al., 2018; Nimura et al., 2009). Furthermore, heterozygous knockout of *Nsd2* in mice impaired T‐ and B‐cell development in an age‐dependent manner (Campos‐Sanchez et al., 2017). These reports suggest that NSD2 plays a fundamental role in cell proliferation and development. However, the role of NSD2 in cellular senescence remains unknown.

Here, we performed an RNAi‐based screen to identify chromatin regulators that affect metabolic and epigenomic functions and found that loss of NSD2 increased mitochondrial mass and oxidative phosphorylation and induced senescence in normal human fibroblasts. Gene expression analyses revealed that loss of NSD2 inhibited cell cycle progression via the RB‐mediated pathway. Chromatin immunoprecipitation (ChIP) and sequencing analyses revealed that NSD2 bound the gene bodies of actively transcribed genes and maintained the levels of H3K36me3. Our data shed light on the epigenomic role of NSD2 in preventing cellular senescence.

## 2 RESULTS

### 2.1 RNAi‐based screen revealed that loss of NSD2 induces cellular senescence

**FIGURE 1:**
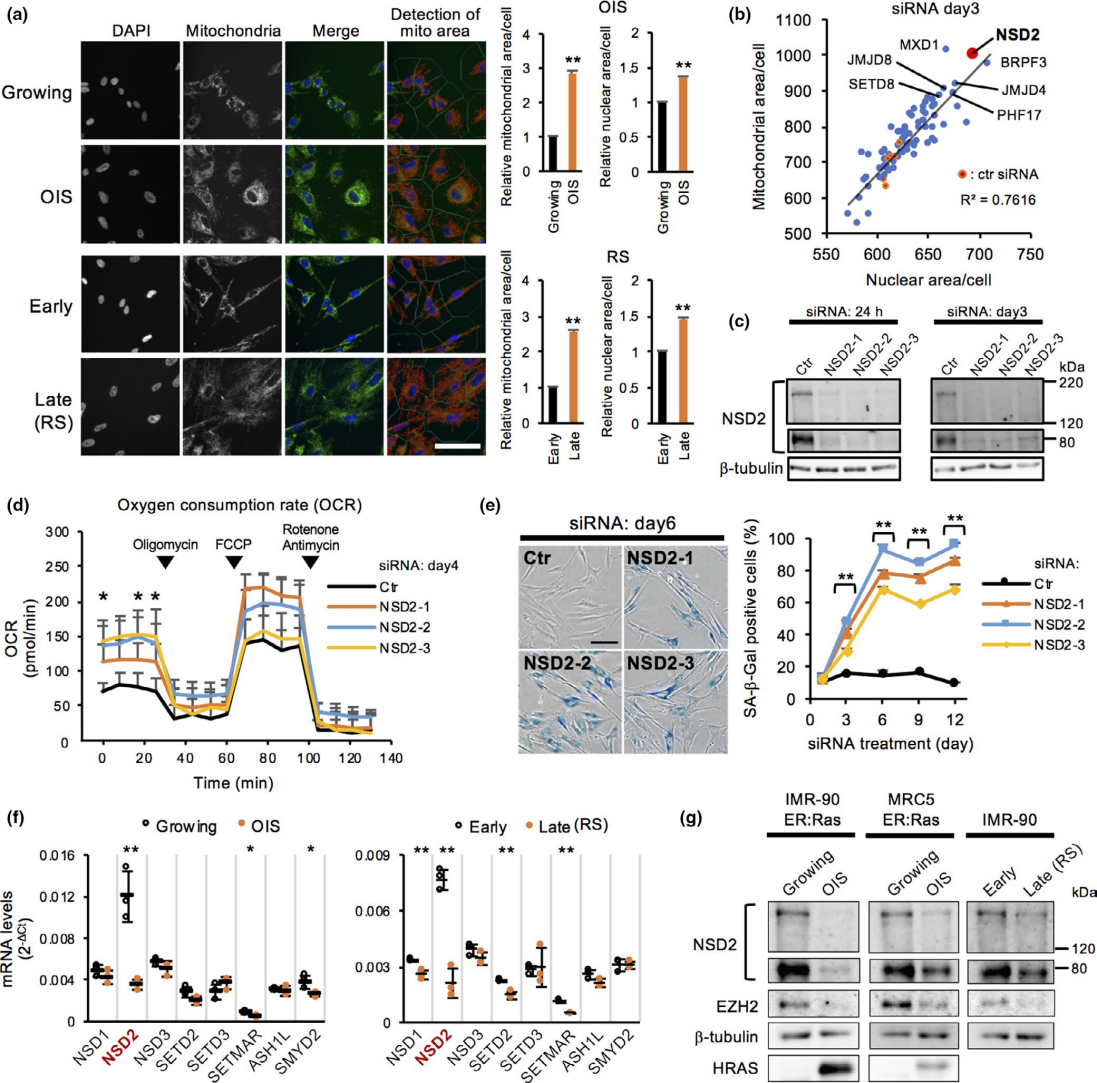
RNAi‐based screen revealed that loss of NSD2 induces mitochondrial activation and cellular senescence. (a) Immunofluorescence of mitochondria in IMR‐90 human fibroblasts with oncogene‐induced senescence (OIS) and replicative senescence (RS). OIS cells were induced by treating IMR‐90 ER:Ras cells with 100 nM 4‐OHT for 6–8 days. RS cells were prepared by repeated passages for 10–12 weeks (Tanaka et al., 2017). To assess mitochondrial signals, the total area of fluorescence signals per cell was calculated in growing, OIS, early passaged, and late passaged (RS) cells (each *n* > 600 cells). Data are shown as means ± *SD*; *n* = 3. Scale bar, 100 μm. (b) Scatter plot between mean mitochondrial area and mean nuclear area in each knockdown cell. Data are shown as means ± *SD*; *n* = 3. (c) Western blot analysis of NSD2 at 24 hr and day 3 in Ctr‐ and NSD2‐KD IMR‐90 cells. (d) OCR on day 4 in Ctr‐ and NSD2‐KD IMR‐90 cells. Data are shown as means ± *SD*; *n* = 4. Respiratory chain inhibitors were serially added to the culture at the indicated time points. Statistical analysis was performed between control siRNA and each NSD2 siRNA. (e) SA‐β‐Gal staining on days 1–12 in Ctr‐ and NSD2‐KD IMR‐90 cells (each *n* > 300 cells). Data are shown as means ± *SD*; *n* = 3. Scale bar, 100 μm. Statistical analysis was performed between control siRNA and each NSD2 siRNA. (f) qRT‐PCR of *NSD2* and other known H3K36 methyltransferases in growing, OIS, early passaged, and late passaged (RS) IMR‐90 cells. Data are shown as means ± *SD*; *n* = 3. (g) Western blot analysis of NSD2 in growing, OIS, early passaged, and late passaged (RS) IMR‐90 and MRC5 human fibroblasts. *p < .05, **p < .01, using Student's *t* test

Senescent cells exhibit active metabolic remodeling characterized by increases of mitochondrial content and oxygen consumption compared with cells in the proliferating state (Takebayashi et al., 2015; Wiley & Campisi, 2016). Using high content imaging analysis, we first confirmed the senescent phenotypes, an increase of mitochondrial and nuclear areas, in human IMR‐90 fibroblasts undergoing oncogenic H‐RAS^G12V^‐induced senescence (OIS) and replicative senescence (RS) (Figure 1a). We then performed an RNA interference (RNAi)‐based screen in IMR‐90 cells using a custom siRNA library against 79 chromatin‐related factors that were predicted to have mitochondrial implications due to the existence of mitochondrial targeting signals and subcellular localization of proteins shown by published databases (Barbe et al., 2008; Claros & Vincens, 1996; Elstner, Andreoli, Klopstock, Meitinger, & Prokisch, 2009; Emanuelsson, Brunak, von Heijne, & Nielsen, 2007; Horton et al., 2007; Pagliarini et al., 2008). We found that knockdown of 23 genes significantly increased mitochondrial area while knockdown of 3 genes significantly decreased it (Table S3). Among the identified factors, SETD8 was previously shown to control senescent processes and senescence‐associated metabolic remodeling by our group and another study (Shih et al., 2017; Tanaka et al., 2017). Notably, transfection of siRNA targeting NSD2 significantly augmented both mitochondrial and nuclear areas within a single cell compared with control siRNAs (ctr) (Figure 1b, Figure S1a). Using three independent siRNAs, we confirmed an increase of mitochondrial content, nuclear area, and mitochondrial oxygen consumption rate (OCR) in NSD2 knockdown (NSD2‐KD) cells compared with those in control knockdown (Ctr‐KD) cells (Figure 1c,d, Figure S1b‐e). Both long and short isoforms of NSD2 were decreased by each knockdown (Figure 1c), whose short isoform lacks the SET domain that is required for histone methyltransferase activity. NSD2‐KD cells showed reduced proliferative activities, as indicated by the reduction of cell number and 5‐ethynyl‐2′‐deoxyuridine (EdU) incorporation starting on day 3 after siRNA transfection (Figure 1f,g). Cell cycle analysis by propidium iodide staining revealed that the population of cells in G2/M phase was slightly increased on day 6 in NSD2‐KD cells (Figure S1h). Furthermore, NSD2‐depleted cells exhibited SA‐β‐Gal staining starting on day 3 after siRNA transfection (Figure 1e, Figure S1i). Loss of NSD2 also inhibited proliferation and increased the number of SA‐β‐Gal‐positive cells in other human fibroblast (Tig‐3) cells (Figure S1j,k). Further, knockdown of the other top‐ranked genes in our screen showed marked senescence features such as reduced EdU incorporation and increased SA‐β‐Gal staining (Figure S1l,m). Collectively, our RNAi‐based high content screen revealed that these genes such as *NSD2* are important to prevent senescence in human fibroblasts.

To further demonstrate the involvement of NSD2 in senescence, we performed qRT‐PCR and Western blot analyses to investigate the levels of NSD2 mRNA and protein expression in senescent cells. We found that NSD2 was downregulated at the mRNA and protein levels in both OIS and RS cells, while the mRNA levels of other known histone methyltransferases against H3K36, such as NSD1 and NSD3, were unchanged or only modestly downregulated (Figure 1f,g, Figure S1n). We next performed overexpression of NSD2 to test whether overexpressed NSD2 acts to prevent senescence. As a result, overexpression of NSD2 did not affect the levels of the induction of SA‐β‐Gal and p16 expression in OIS cells, suggesting that gain of function of NSD2 is not sufficient to prevent senescence (Figure S1o,p,q).

### 2.2 Loss of NSD2 downregulates the expression of cell cycle‐associated genes and DNA repair genes

**FIGURE 2:**
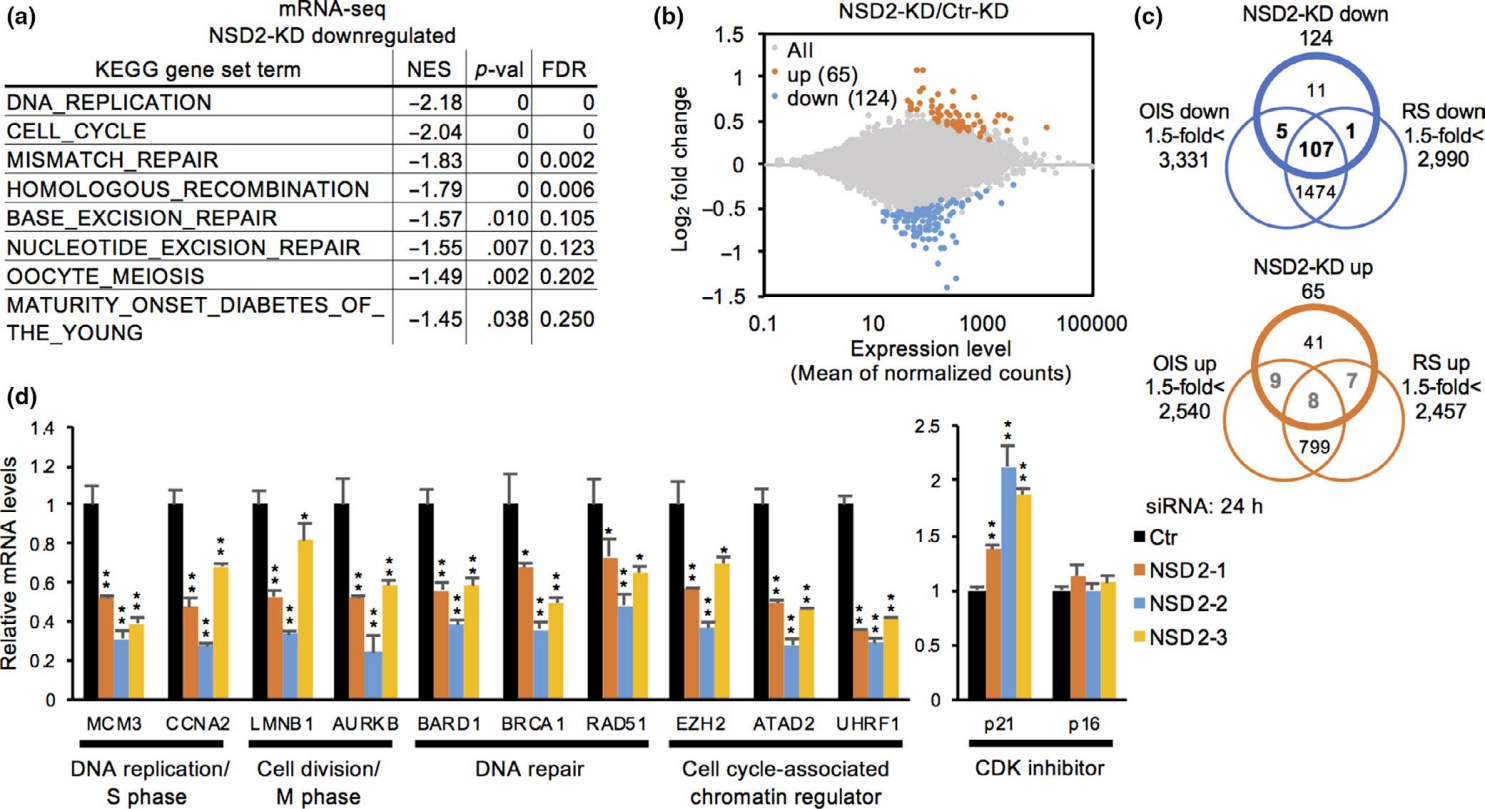
Loss of NSD2 downregulates the expression of cell cycle‐associated genes and DNA repair genes. (a) Gene set list of downregulated genes at 24 hr in NSD2‐KD IMR‐90 cells compared with Ctr‐KD cells. (b) MA plot of differentially expressed genes at 24 hr in NSD2‐KD IMR‐90 cells compared with Ctr‐KD cells. (c) Venn diagrams of commonly downregulated and upregulated genes between NSD2‐KD, OIS, and RS IMR‐90 cells. Transcriptome data of OIS and RS cells were obtained from GSE86546. (d) qRT‐PCR of representative cell cycle‐associated genes and DNA repair genes at 24 hr in Ctr‐ and NSD2‐KD IMR‐90 cells. Data are shown as means ± *SD*; *n* = 3. *p < .05, **p < .01, calculated using Student's *t* test

To elucidate the role of NSD2 in protecting cells from senescence, we performed mRNA‐seq in NSD2‐KD IMR‐90 cells at 24 hr after siRNA transfection. Gene set enrichment analysis (GSEA) using the KEGG platform revealed that eight gene sets were significantly downregulated in NSD2‐KD cells, while no gene set was upregulated (Figure 2a, Figure S2a). The downregulated gene sets included DNA replication, cell cycle, mismatch repair, homologous recombination, base excision repair, and nucleotide excision repair. Analysis of differentially expressed genes by the DESeq2 algorithm identified 124 downregulated and 65 upregulated genes in NSD2‐KD cells (Figure 2b). As shown in unbiased GSEA analysis, the downregulated genes in NSD2‐KD cells were highly enriched for cell cycle‐associated genes and DNA repair genes (Figure S2b). Comparison of transcriptome data between NSD2‐KD, OIS, and RS cells revealed that ~90% (112/124 for OIS and 108/124 for RS) of the downregulated genes and only ~30% (17/65 for OIS and 15/65 for RS) of the upregulated genes in NSD2‐KD cells were consistently upregulated and downregulated in OIS or RS cells, respectively (Figure 2c). qRT‐PCR confirmed the downregulation of cell cycle‐associated genes and DNA repair genes after 24 hr in NSD2‐KD cells (Figure 2d). We also detected the upregulation of *CDKN1A/p21* after 24 hr, and the levels of *CDKN2A/p16* increased after 6 days in NSD2‐KD cells (Figure 2d, Figure S2c). In addition, despite the downregulation of DNA repair genes, NSD2‐KD cells did not elevate the levels of DNA damage, as indicated by levels of DNA damage‐responsive γH2AX foci formation at 24 hr in NSD2‐KD cells, using the treatment with a topoisomerase I inhibitor camptothecin as a positive control (Figure S2d). Consistent with these results, the expression levels of SASP genes were not mostly increased in NSD2‐KD cells, compared with those in SETD8‐KD and OIS cells (Figure S2e). These data indicate that loss of NSD2 decreases the expression of cell cycle‐associated genes and DNA repair genes without inducing massive DNA damage and SASP gene expression.

### 2.3 NSD2 protein is enriched at the gene bodies of actively transcribed genes

**FIGURE 3:**
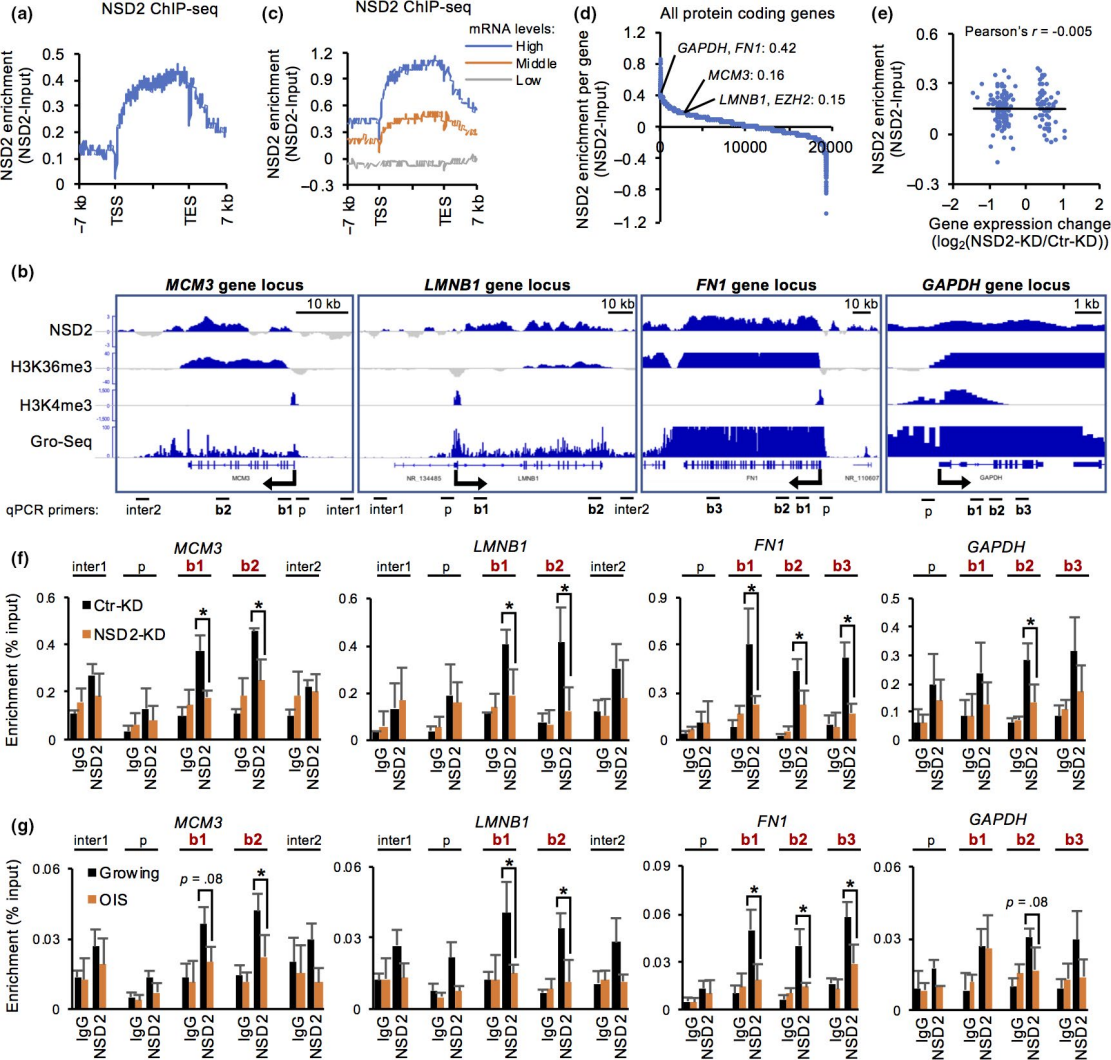
NSD2 is enriched at the gene bodies of actively transcribed genes. (a) Distribution of NSD2 around gene loci in proliferating IMR‐90 cells. (b) Integrative Genomics Viewer tracks showing distribution of NSD2 in *MCM3*, *LMNB1*, *FN1*, and *GAPDH* gene loci. H3K36me3 and H3K4me3 data were obtained from ENCODE datasets. Global Run‐On (GRO)‐seq data were obtained from GSE43070. (c) Distribution of NSD2 around gene loci according to the gene expression levels. High, 100 < base mean; middle, 1 < base mean < 100; and low, base mean < 1 in DESeq2 analysis. (d) Enrichment values of NSD2 in protein‐coding gene bodies. The values were calculated as reads per kilobase transcript per million mapped reads. (e) Scatter plot showing a correlation between enrichment values of NSD2 and expression change of differentially expressed genes in NSD2‐KD IMR‐90 cells. (f) ChIP‐qPCR of NSD2 at intergenic (inter), promoter (p), and gene body (b) of indicated gene loci at 24 hr in Ctr‐ and NSD2‐KD IMR‐90 cells. Primers used are shown in (b). Data are shown as means ± *SD*; *n* = 3. (g) ChIP‐qPCR of NSD2 at intergenic (inter), promoter (p), and gene body (b) of indicated gene loci in growing and OIS IMR‐90 cells. Primers used are shown in (b). Data are shown as means ± *SD*; *n* = 3. *p < .05, **p < .01, calculated using Student's *t* test

To identify the epigenomic contribution and target genes of NSD2, we performed ChIP‐seq using antibodies targeting NSD2 in proliferating IMR‐90 cells (Figure S2f). NSD2 was remarkably enriched at the gene bodies and preferentially at the 3′ region rather than at the transcriptional start site (TSS) and 5′ region of genes (Figure 3a,b). In combination with our mRNA‐seq data, we found that NSD2 was enriched at highly expressed genes compared with genes expressed at low levels (Figure 3c). Further, 60% (11,597/19,473) of all protein‐coding genes appeared to be positively enriched with NSD2, suggesting that NSD2 is widely distributed to actively transcribed, protein‐coding genes (Figure 3d, Figure S2g). Notably, the enrichment of NSD2 was not simply correlated with the gene expression changes observed in NSD2‐KD cells (Pearson's *r* = −.005) (Figure 3e). This result suggested that there is another mechanism that changed the expression levels of NSD2‐target genes (as shown in Figure 5).

Indeed, we confirmed the enrichment of NSD2 at the gene bodies of both downregulated genes (*MCM3*, *LMNB1*, and *EZH2*) and stable high‐expression genes (*FN1* and *GAPDH*) (Figure 3f, Figure S3a,b). Furthermore, these enrichments were decreased in NSD2‐KD cells. We also observed a decrease of NSD2 enrichment at these gene loci in OIS cells (Figure 3g). Our data suggest that NSD2 is enriched at the gene bodies of actively transcribed genes possibly for maintaining their expression activities.

### 2.4 Loss of NSD2 affects the levels of H3K36 trimethylation at NSD2‐enriched gene bodies

**FIGURE 4:**
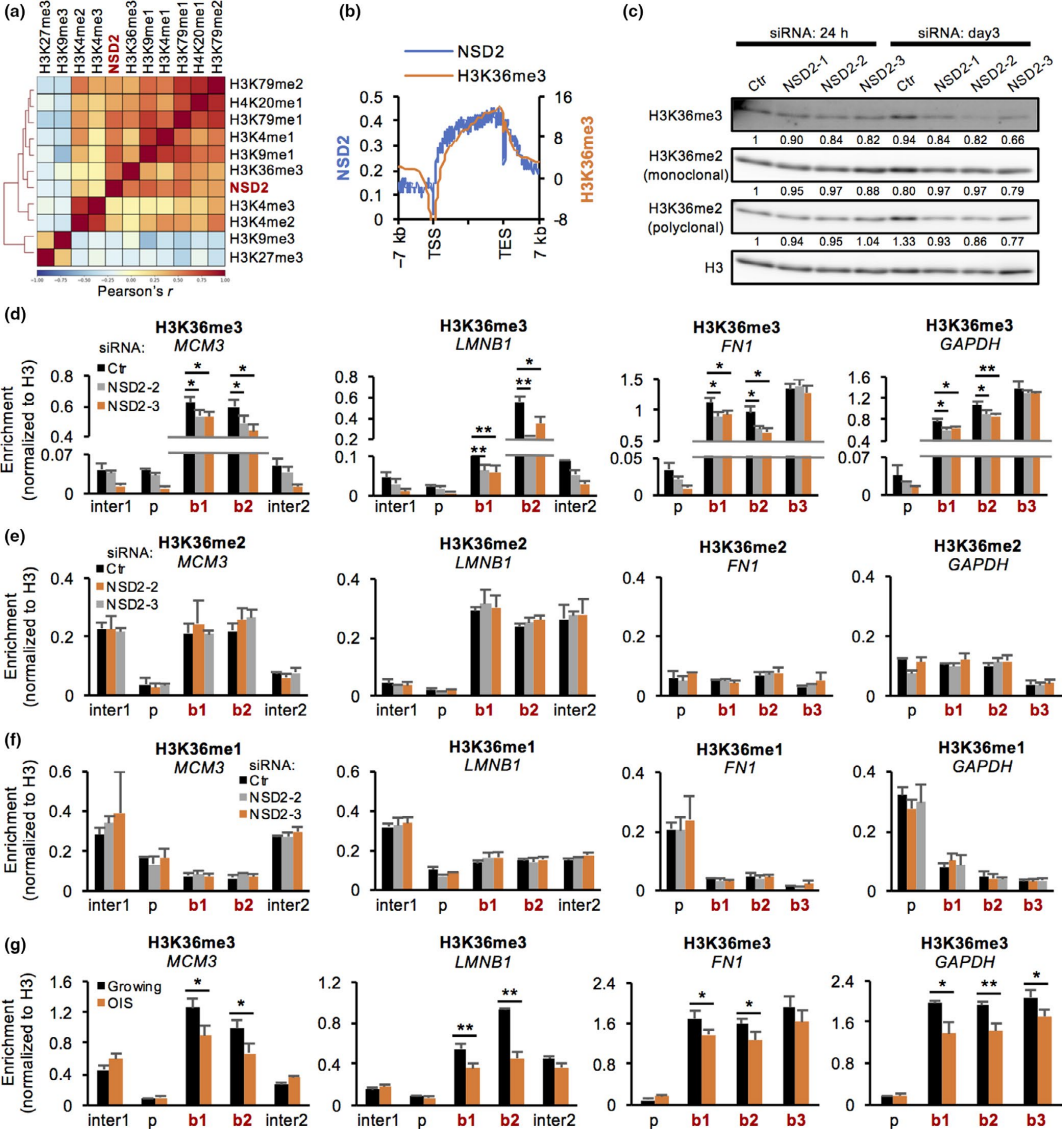
Loss of NSD2 alters the levels of H3K36 trimethylation at NSD2‐enriched gene bodies. (a) Heatmap showing a genome‐wide correlation between NSD2 and histone methylations in IMR‐90 cells. Histone modification data were obtained from ENCODE datasets. (b) Distribution of NSD2 and H3K36me3 around gene loci. (c) Western blot analysis of H3K36me3 and H3K36me2 at 24 hr and day 3 in Ctr‐ and NSD2‐KD IMR‐90 cells. The intensity of bands was calculated using ImageJ and normalized to H3 and then to Ctr‐KD of 24 hr. (d–f) ChIP‐qPCR of H3K36me3, me2, or me1 at intergenic (inter), promoter (p), and gene body (b) of indicated gene loci at 24 hr in Ctr‐ and NSD2‐KD IMR‐90 cells. Primers used are shown in Figure 3b. Data are shown as means ± *SD*; *n* = 3. (g) ChIP‐qPCR of H3K36me3 at intergenic (inter), promoter (p), and gene body (b) of indicated gene loci in growing and OIS IMR‐90 cells. Primers used are shown in Figure 3b. Data are shown as means ± *SD*; *n* = 3. *p < .05, **p < .01, *p*‐values were calculated using Student's *t* test

By comparison with ENCODE datasets of histone modifications in IMR‐90 cells, we found that the enrichment of NSD2 was positively correlated with transcriptionally active, gene body‐enriched marks, such as H3K36me3, H3K4me1, H3K9me1, H4K20me1, and H3K79me1, and poorly linked with repressive marks, such as H3K27me3 and H3K9me3 (Figure 4a, Figure S3c). Among these marks, H3K36me3 was highly correlated with NSD2 in terms of the preferential distribution at the 3′ region of the gene bodies (Figure 4b, Figure S3d). This is consistent with the role of NSD2 as a methyltransferase and a reader protein for H3K36me3 (Nimura et al., 2009; Vermeulen et al., 2010). To test whether loss of NSD2 alters the levels of H3K36 methylation at NSD2‐target gene loci, we performed Western blot, immunofluorescence, and ChIP‐qPCR analyses for H3K36 methylation marks. The total amounts of H3K36me3 and H3K36me2 did not change in NSD2‐KD cells at 24 hr and slightly decreased on day 3 (Figure 4c). However, ChIP‐qPCR revealed that the levels of H3K36me3 were significantly decreased at the gene body of both downregulated genes (*MCM3* and *LMNB1*) and stably transcribed genes (*FN1* and *GAPDH*) at 24 hr in NSD2‐KD cells (Figure 4d). In contrast, the levels of H3K36me2 and H3K36me1 were not changed at NSD2‐target gene loci in NSD2‐KD cells (Figure 4e,f). We further confirmed the stability of H3K36me2 levels using other antibodies (Figure S3e,f). We also confirmed a decrease of H3K36me3 levels at NSD2‐target gene loci in OIS cells, while the levels of H3K36me2 and H3K36me1 were not changed or even increased at these loci (Figure 4g, Figure S3g,h,i). Collectively, these data suggest that NSD2 is involved in maintenance of the levels of H3K36me3 at NSD2‐target gene bodies.

### 2.5 Loss of NSD2 downregulates the promoter activities of RB‐associated genes

**FIGURE 5:**
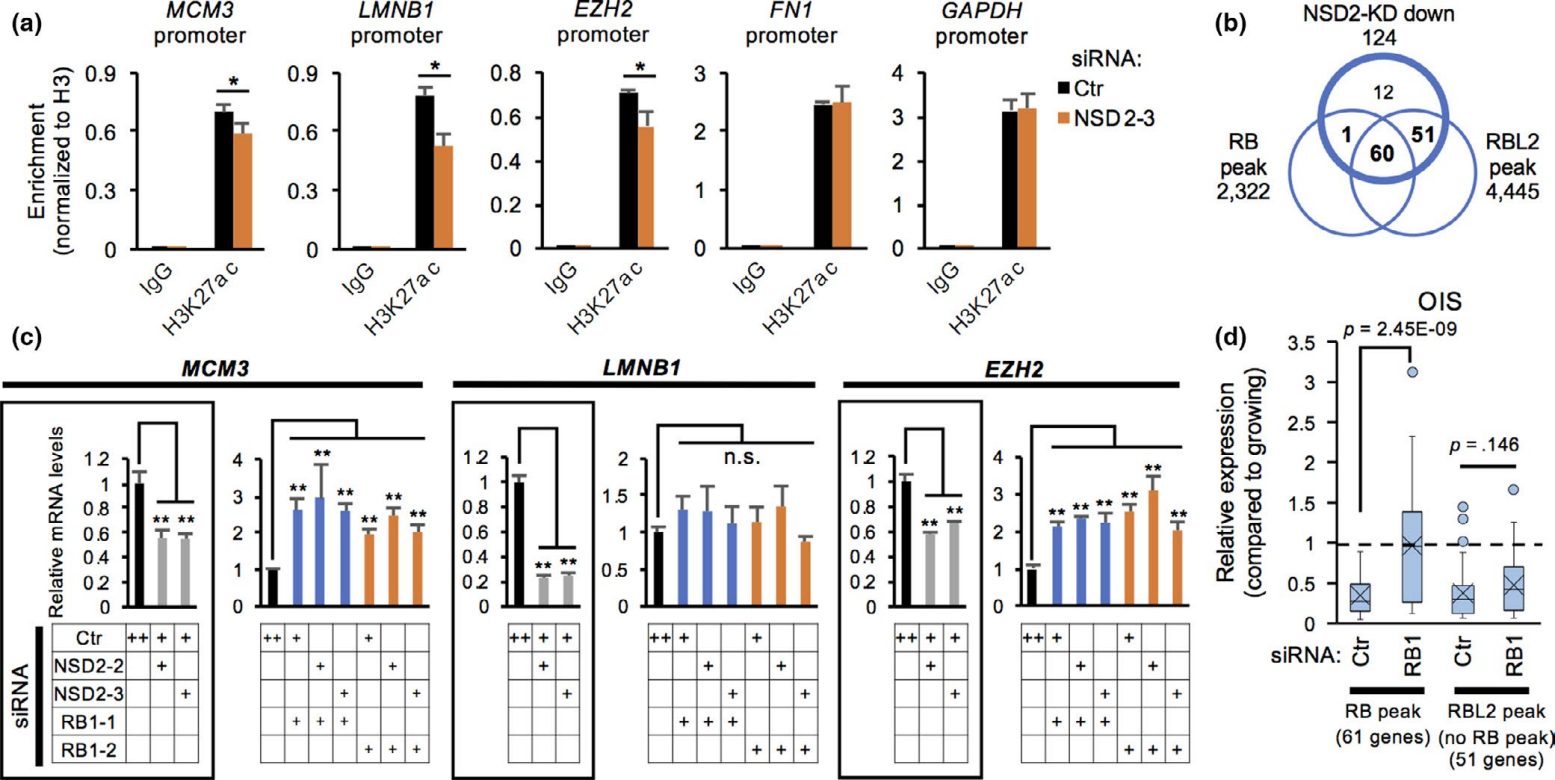
Loss of NSD2 downregulates the promoter activities of RB‐associated genes. (a) ChIP‐qPCR of H3K27 acetylation (ac) at promoters of indicated gene loci in Ctr‐ and NSD2‐KD IMR‐90 cells. Data are shown as means ± *SD*; *n* = 3. (b) Venn diagram of genes downregulated in NSD2‐KD IMR‐90 cells, RB‐associated genes, and RBL2‐associated genes. (c) qRT‐PCR of representative RB‐associated, cell cycle‐associated genes on day 2 in Ctr‐, NSD2‐, and RB1‐KD IMR‐90 cells. Data are shown as means ± *SD*; *n* = 3. (d) Relative expression levels of RB‐ or RBL2‐associated, NSD2‐KD downregulated genes in Ctr‐ and RB1‐KD OIS IMR‐90 cells. Data were obtained from GSE60652. *p < .05, **p < .01, calculated using Student's *t* test

To investigate the specificity of expression changes in NSD2‐target genes (Figures 3 and 4), we examined the promoters of NSD2‐target genes. Interestingly, loss of NSD2 resulted in decreased levels of H3K27 acetylation (ac) at the promoter region of the downregulated genes such as *MCM3*, *LMNB1*, and *EZH2* at 24 hr after siRNA transfection, but no changes were observed at *FN1* and *GAPDH* genes (Figure 5a). To identify the specific chromatin regulators at the promoter region of genes downregulated in NSD2‐KD cells, we further analyzed public ChIP‐seq datasets using ChIP‐Atlas (Oki et al., 2018). The promoter region of the downregulated genes in NSD2‐KD cells was highly enriched by E2F4, RBL2, and E2F1 (Figure S4a). We found that 41% (61/124) and 90% (111/124) of the downregulated genes were targeted by RB and RBL2, respectively (Figure 5b, Figure S4b) (Chicas et al., 2010). To examine whether the decreased expression of cell cycle‐associated genes in NSD2‐KD cells involves RB or RBL2, we performed simultaneous knockdown of NSD2 and RB or RBL2. Knockdown of RB completely abolished the reduced expression of RB‐associated genes such as *MCM3*, *LMNB1*, and *EZH2* in NSD2‐KD cells (Figure 5c, Figure S4c). Likewise, knockdown of RB prevented the reduced expression of RB‐associated, NSD2‐KD downregulated genes during induction of OIS (Figure 5d). In contrast, loss of RB did not fully restore the reduced expression of RBL2‐associated, RB‐nonassociated genes, such as *AURKB* and *CCNA2*, in OIS and NSD2‐KD cells (Figure 5d, Figure S4d). In addition, knockdown of RBL2 did not restore the reduced expression of RBL2‐associated, RB‐nonassociated genes in NSD2‐KD cells (Figure S4e). Interestingly, knockdown of RB decreased the amount of SA‐β‐Gal in NSD2‐KD cells (Figure S4f). These results indicate that loss of NSD2 downregulates cell cycle‐associated genes and promotes senescence at least in part via the RB‐mediated pathway.

### 2.6 NSD2 is controlled in a cell cycle‐dependent manner and is required for expression of late serum response genes

**FIGURE 6:**
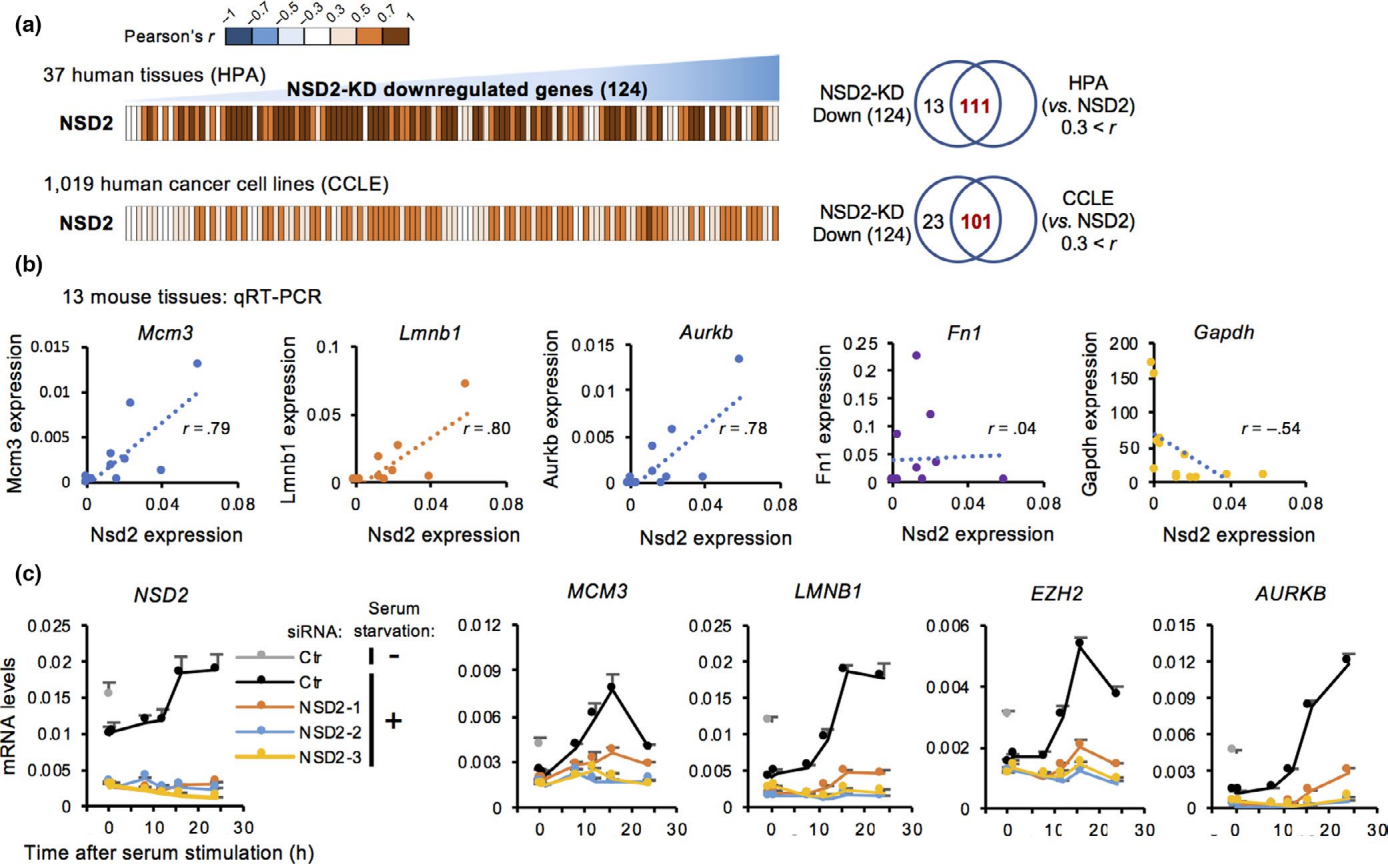
NSD2 is controlled in a cell cycle‐dependent manner and is required for the expression of late serum response genes. (a) Heatmaps showing the correlation between mRNA expression levels of *NSD2* and genes downregulated in NSD2‐KD IMR‐90 cells in 37 human normal tissues and 1,019 human cancer cell lines. Data were obtained from the Human Protein Atlas (HPA) and Cancer Cell Line Encyclopedia (CCLE). Venn diagrams showing the number of positively correlated genes among genes downregulated in NSD2‐KD IMR‐90 cells in HPA or CCLE. (b) Scatter plots showing the correlation of gene expression between *Nsd2* and *Mcm3*, *Lmnb1*, *Aurkb*, *Fn1*, or *Gapdh* in 13 normal tissues from 7‐week‐old male mice. Data are shown as means ± *SD*; *n* = 3. (c) qRT‐PCR of *NSD2*, *MCM3*, *LMNB1*, *EZH2*, and *AURKB* during serum stimulation in IMR‐90 cells. *36B4* was used for normalization. Data are shown as means ± *SD*; *n* = 3. *p < .05, **p < .01, calculated using Student's *t* test

NSD2 is highly expressed in several types of cancers, and depletion of NSD2 causes growth retardation during development in mice (Nimura et al., 2009; Vougiouklakis et al., 2015). To elucidate the physiological role of NSD2, we analyzed the correlation of expression levels between *NSD2* and NSD2‐target genes in 37 human normal tissues and 1,019 human cancer cell lines using the Human Protein Atlas (HPA) and Cancer Cell Line Encyclopedia (CCLE), respectively (Barretina et al., 2012; Uhlén et al., 2015). The expression levels between *NSD2* and the downregulated genes in NSD2‐KD cells were positively correlated in both normal tissues and cancer cell lines (Figure 6a, Figure S5a). We also observed a negative correlation between the levels of *NSD2* and *CDKN1A/p21* in cancer cell lines (Figure S5b,c), whereas most of the upregulated genes in NSD2‐KD cells showed no negative correlation with *NSD2*. We further found a positive correlation between *Nsd2* and cell cycle‐associated genes in mouse normal tissues by qRT‐PCR (Figure 6b). *NSD2* was highly expressed in testis, thymus, seminal vesicle, and spleen and expressed at lower levels in skeletal muscle, skin, and heart (Figure S5d). We further found that the expression of *Nsd2* was decreased in spleen of aged mice compared with levels in young mice (Figure S5e).

The correlated expression levels between *NSD2* and cell cycle‐associated genes indicate that the expression of *NSD2* is regulated under growth signaling. To examine the expression of *NSD2* during cell cycle progression, we used serum stimulation after serum starvation in IMR‐90 cells (Figure S5f). Notably, the levels of *NSD2* were remarkably decreased in serum‐starved, quiescent cells compared with cells in growing conditions (Figure 6c). Furthermore, *NSD2* was upregulated after serum addition (during S and G2 phases) in parallel with cell cycle‐associated genes. Importantly, knockdown of NSD2 dampened the upregulation of cell cycle‐associated genes without affecting the induction of immediate early genes such as *FOS* and *JUN* (Figure 6c, Figure S5g). Consistent with the mRNA expression levels, NSD2 protein was enriched during S and G2 phases compared with levels in G1 phase (Figure S5h). In contrast, in RS cells (late), *NSD2* was not further decreased by serum starvation, and serum stimulation did not induce expression of *NSD2* and cell cycle‐associated genes (Figure S5i). Taken together, these results suggest that the expression of *NSD2* is induced by serum stimulation and that NSD2 is required for the induction of cell cycle‐related genes.

## 3 DISCUSSION

Here, we demonstrated that NSD2 plays a pivotal role in preventing senescence‐associated epigenomic remodeling in human fibroblasts through maintaining H3K36me3 levels and RB‐mediated cell cycle regulation. Our initial RNAi screen revealed that loss of NSD2 increased mitochondrial content, which is a hallmark of senescent cells (Wiley & Campisi, 2016). Consistent with this finding, depletion of NSD2 increased mitochondrial OCR and eventually induced cellular senescence (Figure 1). Transient depletion of NSD2 was sufficient to reduce the expression levels of cell cycle‐associated genes, at least in part by the RB‐mediated pathway (Figures 2 and 5). We previously reported that loss of another histone methyltransferase SETD8 activated mitochondrial respiration in a RB‐dependent manner during senescence in human fibroblasts (Tanaka et al., 2017). Knockdown of RB abolished mitochondrial activation during OIS (Takebayashi et al., 2015). Here, we showed that loss of NSD2 also contributes to the remodeling of mitochondrial activities during senescence by reinforcing RB function. Together, our data revealed a novel link between NSD2 and RB in preventing senescent transition and senescence‐associated metabolic remodeling.

How loss of NSD2 activates RB function remains unclear. RB is activated by p21 and p16 due to inhibition of the cyclin‐dependent kinases (Muñoz‐Espín & Serrano, 2014). Transient depletion of NSD2 increased the expression of *p21* gene (Figure 2d), and the expression of *p16* was subsequently augmented in NSD2‐KD cells (Figure S2c). Thus, loss of NSD2 may activate RB by inducing p21 and p16, although it is still unclear how transient depletion of NSD2 induced *p21*. The expression of *p21* is controlled by various mechanisms including p53 transactivation and DNA damage response in senescence (Muñoz‐Espín & Serrano, 2014). Notably, there was no marked increase in DNA damage signals manifested by accumulation of γH2AX and SASP gene expression in NSD2‐KD cells (Figure S2d,e). In contrast, a previous report suggested that NSD2 can regulate p53 protein stability by adding methylation on Aurora kinase A (AURKA) (Park, Chae, Kim, Oh, & Seo, 2018). Methylated AURKA interacts with p53 and accelerates proteasome‐mediated degradation of p53. Thus, depletion of NSD2 might stabilize p53 via loss of methylation of AURKA, resulting in *p21* induction. NSD2 was also dynamically induced during S and G2 phases and was essential for the induction of cell cycle‐associated genes followed by serum stimulation (Figure 6c, Figure S5h). Taken together, our data suggest that NSD2 acts as a cell cycle regulator by cooperating with p53, p21, and RB. Interestingly, the overexpression of NSD2 did not affect the levels of the induction of SA‐β‐Gal and *p16* expression in OIS cells. Further studies are required to clarify whether the NSD2 contributes to the prevention of senescence via methylation of nonhistone proteins.

NSD2 was preferentially enriched at the gene bodies of actively transcribed genes, concordant with the enrichment of H3K36me3 in human fibroblasts (Figure 3). The correlation of NSD2 and H3K36me3 was also observed by ChIP‐seq in K562 human leukemia cells (Ram et al., 2011). Notably, NSD2 directly binds H3K36me3 possibly via its PHD or PWWP domain (Sarai et al., 2013; Vermeulen et al., 2010). We observed a significant decrease of H3K36me3 levels at the NSD2‐enriched gene bodies including at stably expressed genes in NSD2‐KD cells (Figure 4). Similarly, the levels of H3K36me3 were decreased at these loci in OIS cells. NSD2 was previously reported to be capable of adding trimethylation on H3K36 (Nimura et al., 2009). Furthermore, loss of NSD2 caused a reduction of H3K36me3 levels at its target gene bodies (Martinez‐Garcia et al., 2011; Sarai et al., 2013; Yang et al., 2012). Although the capability of NSD2 to directly confer trimethylation on H3K36 is still controversial (Kuo et al., 2011; Li et al., 2009; Poulin et al., 2016), our ChIP and ChIP‐seq data strongly suggest that NSD2 is required for the maintenance of H3K36me3 levels at the gene bodies of actively transcribed genes by directly acting on the chromatin. Notably, previous evidence suggested that NSD2 might contribute to the persistence of pre‐existing H3K36me3 levels rather than the establishment of new H3K36me3 at interferon response genes (Sarai et al., 2013). While many reports state that NSD2 confers mono‐ or di‐methylation on H3K36 (Kuo et al., 2011; Li et al., 2009; Poulin et al., 2016), we did not observe any decrease of H3K36me1 or H3K36me2 levels at NSD2‐enriched gene bodies in both NSD2‐KD cells and OIS cells (Figure 4e,f, Figure S3f,g,h,i). Indeed, NSD2‐overexpressing cancer cells accumulated H3K36me2 preferentially at genomic intergenic regions rather than gene bodies (García‐Carpizo et al., 2016; Popovic et al., 2014). Given that the expression levels of NSD2 are dynamically regulated during the cell cycle (Evans et al., 2016) and that NSD2 is overexpressed in various types of cancer cells (Vougiouklakis et al., 2015), the role of NSD2 might vary in cell cycle‐ and dose‐dependent manners as well as in a genomic locus‐specific manner.

Misregulation of H3K36me3 is one of the hallmarks in aged model organisms (Pu et al., 2015; Sen et al., 2015; Wood et al., 2010). We observed a marked change in expression levels at only a part of the NSD2‐enriched genes in NSD2‐KD cells (Figures 2 and 3). Therefore, what is the biological significance of H3K36me3 maintenance by NSD2? H3K36me3 is recognized by DNA methyltransferase 3B (DNMT3B) and protects genes from spurious RNA polymerase II entry and cryptic transcription initiation (Neri et al., 2017). Furthermore, DNA repair‐associated proteins, such as human MutS homolog 6 (hMSH6) and lens epithelium‐derived growth factor (LEDGF), interact with H3K36me3 and facilitate DNA repair at gene bodies (Aymard et al., 2014; Daugaard et al., 2012; Li et al., 2013; Pfister et al., 2014). p52, a short isoform of LEDGF, and MORF‐related gene 15 (MRG15) also bind H3K36me3 and mediate alternative splicing (Luco et al., 2010; Pradeepa, Sutherland, Ule, Grimes, & Bickmore, 2012). Thus, there is the possibility that NSD2 functions for epigenomic maintenance and gene regulation to protect from cellular senescence by preserving H3K36me3 levels.

Global correlation of gene expression levels between NSD2 and the cell cycle‐associated genes in various tissues and cancer cell lines suggested that NSD2 is implicated in cell cycle regulation in diverse cell types (Figure 6). Indeed, haploinsufficiency of *Nsd2* gene in mice resulted in developmental growth retardation (Nimura et al., 2009) and defects in long‐term maintenance of B and T lymphocytes during aging (Campos‐Sanchez et al., 2017). Interestingly, we also observed a decrease of *Nsd2* expression levels in aged spleen tissue in mice (Figure S5e). These observations emphasized the importance of precise control of NSD2 expression to protect cells from aging as well as cancer.

In summary, our results show that NSD2 has an epigenomic role together with RB: NSD2 maintains H3K36me3 at the bodies of actively transcribed genes and cell cycle‐related genes to prevent cellular senescence.

## 4 EXPERIMENTAL PROCEDURES

Full experimental procedures are included in the Supporting Information.

### 4.1 ChIP‐qPCR and ChIP‐seq analyses

For ChIP‐qPCR analysis, cells were crosslinked with PBS containing 1% formaldehyde for 10 min. Cells were lysed, and the lysates were sonicated using a Bioruptor (Cosmo Bio) with 10–30 sonications of 30 s each with 30 s intervals. Sonicated samples were then incubated with 2–4 μg of each antibody at 4°C overnight, followed by pull‐down assay using protein A/G‐conjugated agarose beads (Millipore). After decrosslinking and RNase and Proteinase K treatments, DNA was extracted by phenol‐chloroform extraction and subjected to qPCR using the primers listed in Table S1.

For genome‐wide NSD2 distribution analysis, sonicated samples were incubated with antibodies conjugated with a Dynabeads M‐280 sheep anti‐mouse IgG (Invitrogen, 11201D) at 4°C overnight, followed by pull‐down assay using a magnetic stand. Extracted DNA was subjected to adaptor ligation using the NEBNext Ultra II DNA Library Prep Kit for Illumina (New England Biolabs). Sequencing was performed on a NextSeq 500 (Illumina) with 75‐bp single‐end reads, and data analyses were performed on the Galaxy platform. The reads were trimmed using Trimmomatic v.0.36.3 and mapped to the hg19 reference genome using BWA v.0.7.15.1. After removing duplicate reads using Picard MarkDuplicates v.1.136.0, the reads were normalized to those of input by deepTools bamCompare v.2.5.0.0 and visualized with an Integrative Genomics Viewer. Distributions around gene loci were calculated and visualized using deepTools computeMatrix and plotProfile, respectively. The number of reads in each gene was calculated by featureCounts v.1.4.6.p5. For correlation analyses between NSD2 and histone modifications, the reads were calculated with deepTools multiBigwigSummary at 10 kb bin size and visualized with plotCorrelation. The peak detection of RB and RBL1 was performed by MACS v.1.0.1. All histone modification ChIP‐seq data in IMR‐90 cells were obtained from the ENCODE project (https://www.encodeproject.org) (Consortium, 2012). RB and RBL2 ChIP‐seq data in IMR‐90 cells were obtained from GSE19899 (Chicas et al., 2010). Global Run‐On (GRO)‐seq data were obtained from GSE43070 (Jin et al., 2013). The ChIP‐seq data were deposited in the GEO database under accession code GSE138067.

### 4.2 Assessment of mitochondrial activities

Real‐time monitoring of cellular OCR was performed by a XF24 extracellular flux analyzer (Seahorse Bioscience) as previously described (Tanaka et al., 2017). For determination of mitochondrial mass, cells were stained with 5 μg/ml JC‐1 in culture medium for 30 min at 37°C, followed by flow cytometric analysis.

## CONFLICT OF INTEREST

None declared.

## AUTHOR CONTRIBUTIONS

H.T., T.I., K.E., T.K., S.T., and M.N. designed and conducted the experiments. H.T., T.I., T.K., and M.N. prepared the manuscripts.

## Supporting information

Supplementary Material

Table S3

## Data Availability

The data that support the findings of this study are openly available in GEO at [https://www.ncbi.nlm.nih.gov/geo/], reference numbers [GSE86546, GSE43070, GSE60652, GSE19899, and GSE16256].
