# Supplementary material for: The NSD2/WHSC1/MMSET methyltransferase prevents cellular senescence‐associated epigenomic remodeling
Source: Aging Cell. 2020 Jun 22;19(7):e13173. doi: 10.1111/acel.13173 (PMC7433007; doi:10.1111/acel.13173)
Supplement: Supplementary file 1 — Supplementary Material [file ACEL-19-e13173-s001.pdf]

Supporting information

The NSD2/WHSC1/MMSET methyltransferase prevents cellular senescence-associated epigenomic remodeling

Authors:

Hiroshi Tanaka, Tomoka Igata, Kan Etoh, Tomoaki Koga, Shin-ichiro Takebayashi, and Mitsuyoshi Nakao

Supporting information contains:

Supporting experimental procedures

Supporting information Figure legends

References

Supporting information Figures S1-S5

Supporting information Tables S1-S4

SUPPORTING EXPERIMENTAL PROCEDURES

Cell culture, siRNA, and mice

IMR90, Tig-3, IMR-90 ER:Ras (H-RasG12V), and MRC5 ER:Ras (H-RasG12V) cells were maintained in Dulbecco's modified Eagle's medium (DMEM)/F12 supplemented with 10% (v/v) heat-inactivated fetal bovine serum (FBS). To induce OIS, IMR-90 ER:Ras or MRC5 ER:Ras cells were treated with 100 nM 4-OHT for 6–8 days. RS cells were prepared by repeated passages for 10–12 weeks (Tanaka et al., 2017). To induce quiescence, cells were cultured in DMEM/F12 supplemented with 0.1% (v/v) FBS for 48 h.

Transfection of siRNA was performed every 3 days using RNAiMAX (Invitrogen). The siRNAs used in this study are listed in **Table S2**. In addition, the statistical significance of each siRNA in the screen was shown in **Table S3**.

Animal experiments were conducted in accordance with the guideline of the Animal Care and Use Committee of Kumamoto University. For gene expression analysis, 7-week-old male C57BL/6J mice were sacrificed and analyses were performed in 14 mouse tissues. For gene expression analysis in young and aged spleen, 12-week-old and 29–31-month-old female C57BL/6J mice were sacrificed.

Reagents

The following reagents were used in this study: JC-1 (Molecular Probes, Eugene, OR, USA), oligomycin, carbonyl cyanide 4-(trifluoromethoxy)phenylhydrazone (FCCP), rotenone, and antimycin A (all from Sigma Aldrich, St. Louis, MO, USA). The antibodies used were as follows: anti-NSD2 (ab75359), anti-H3K36me1 (ab9048), anti-H3K36me2 (polyclonal) (ab9049), anti-H3K36me3 (ab9050), anti-pan H3 (ab1791), anti-mitochondria (ab3298) (Abcam, Cambridge, UK); anti-H3K36me2 (monoclonal) (2901; Cell Signaling Technology, Danvers, MA, USA); anti-H-Ras (sc-29), anti-mouse IgG (sc-2025), anti-rabbit IgG (sc-2027) (Santa Cruz Biotechnology, Dallas, TX, USA); anti-H3K27ac (07-360), anti-phospho-histone H2AX (05-636) (Millipore); anti-EZH2 (612666; BD); anti-actin (A2103), anti-β-tubulin (T4026) (Sigma Aldrich).

Quantitative reverse transcription PCR (qRT-PCR) and mRNA-seq analyses

Total RNA was extracted from cultured cells with TRIzol Reagent (Invitrogen). For qRT-PCR, cDNA was produced using ReverTra Ace qPCR RT Master Mix, followed by qPCR with SYBR green fluorescence using THUNDERBIRD reagent (Toyobo, Osaka, Japan) and a StepOnePlus Real-Time PCR instrument (Thermo Fisher Scientific, Waltham, MA, USA). *GAPDH* was used for normalization unless otherwise noted. Primers used are listed in **Table S1**.

For transcriptome analysis by mRNA-seq, mRNA was purified using the NEBNext Poly(A) mRNA Magnetic Isolation Module (New England Biolabs). For sequencing, cDNA library was synthesized using the NEBNext Ultra RNA Library Prep Kit for Illumina (New England Biolabs). Sequencing was performed on a MiSeq (Illumina) with 150-bp single-end reads and data analyses were performed on the Galaxy platform (http://usegalaxy.org). The reads were trimmed with Trimmomatic v.0.36.3 and the resulting 2.9–3.4 million reads were mapped to the hg19 reference genome using HISAT2 v2.1.0. Uniquely mapped reads were extracted by SAMtools and the numbers of reads were calculated with featureCounts v.1.6.2. Differentially expressed genes were

identified by DESeq2 v.2.11.40.2 between 2 biological replicates of Ctr-KD cells and the sum of each one biological replicate of NSD2-KD cells using either of NSD2-1, NSD2-2, and NSD2-3 siRNA. Gene set annotation analyses were performed by using the GSEA v.3.0 (Subramanian et al., 2005) and the Metascape (Zhou et al., 2019). We showed the gene list of differentially expressed genes (**Table S4**). The mRNA-seq data were deposited in the GEO database under accession code GSE138067.

Western blot analysis

Western blot analysis was performed as previously described (Tanaka et al., 2017).

Cell counting and cell cycle analyses

Cell counting and cell cycle analyses were performed as previously described (Tanaka et al., 2017).

SA-β-Gal staining and EdU incorporation assays

SA-β-Gal staining was performed with the Senescence Detection Kit (BioVision, Milpitas, CA, USA), according to the manufacturer's instruction. The EdU incorporation assay was performed using the Click-iT EdU Alexa Fluor 594 Imaging Kit (Invitrogen) as previously described (Tanaka et al., 2017).

Immunofluorescence and high content imaging analyses

Cells were fixed with 4% paraformaldehyde for 10 min at room temperature. Cells were then permeabilized, blocked with 0.5% BSA, and incubated with primary antibodies for 1 h at room temperature, followed by incubation with Cy3- or Alexa Fluor 488-conjugated secondary antibody for 1 h. DNA was counterstained with 0.5 μg/ml DAPI.
For high content imaging analysis, the images were obtained and analyzed using Cellomics CellInsight with HCS studio cell analysis software (Thermo Fisher Scientific). To quantify the total fluorescence intensity and the total area of fluorescence signals in each cell, the Spot Detector BioApplication was used. Each cell was defined by a DAPI channel.

For RNAi screen, 2,500 cells of IMR-90 cells were seeded with 5 nM of each siRNA in 96 well plate. After 3 days, cells were fixed and subjected to immunofluorescence using anti-mitochondrial antibody (ab3298). Sixteen images per well were taken by using the CellInsight with 20x magnification. Total area of mitochondria and nucleus per cell was calculated using the Spot Detector BioApplication. These screens were performed at three biological replicates, and the hits were defined by the magnitude of change in mitochondrial area and statistical analysis using student's *t*-test between control siRNA and samples.

Overexpression of NSD2

For NSD2 overexpression, the monomeric enhanced GFP (mEGFP)-fused NSD2 (NP_579877), NSD2-EGFP, was inserted into CSII-CMV-MCS-IRES2-Bsd (RDB04385 from RIKEN BRC). IMR-90 ER:Ras cells were transduced and selected for 6 days with 10 μM Blasticidin S (invitrogen, R210-01). OIS was induced with 100 nM 4-OHT for 6 days.

Statistical analysis

Data are presented as means ± s.d. All statistical analyses were performed by two-tailed Student's *t*-test.

SUPPORTING INFORMATION FIGURE LEGENDS

Supporting information Figure 1. Loss of NSD2 induces mitochondrial activation and cellular senescence. Related to Figure 1. (**a**) Immunofluorescence of mitochondria at 72 h in Ctr- and NSD2-KD IMR-90 cells in RNAi screen. (**b**) qRT-PCR of *NSD2* at 24, 72 h and day 6 in Ctr- and NSD2-KD IMR-90 cells. Data are shown as means ± s.d.; n=3. (**c**) Assessment of the mean mitochondrial area measured by high content imaging analysis on day 3 in Ctr- and NSD2-KD IMR-90 cells (each n>1,200 cells). Data are shown as means ± s.d.; n=3. (**d**) Assessment of the mean nuclear area measured by high content imaging analysis on day 3 in Ctr- and NSD2-KD IMR-90 cells (each n>1,200 cells). Data are shown as means ± s.d.; n=3. (**e**) Assessment of mitochondrial mass by the mean fluorescence of JC-1 monomer measured by flow cytometry on day 3 in Ctr- and NSD2-KD IMR-90 cells. Data are shown as means ± s.d.; n=3. (**f**) Growth curves of Ctr- and NSD2-KD IMR-90 cells. Data are shown as means ± s.d.; n=3. Statistical analysis was performed between control siRNA and each NSD2 siRNA. (**g**) EdU incorporation assay on day 3 in Ctr- and NSD2-KD IMR-90 cells (each n>600 cells). Data are shown as means ± s.d.; n=3. (**h**) Cell cycle analysis evaluated by flow cytometry on day 6 in Ctr- and NSD2-KD IMR-90 cells. Data are shown as means ± s.d.; n=3. (**i**) SA-β-Gal staining on day 3 in Ctr- and NSD2-KD IMR-90 cells. Scale bar, 100 μm. (**j**) Growth curves of Ctr- and NSD2-KD Tig-3 cells. Data are shown as means ± s.d.; n=3. Statistical analysis was performed between control siRNA and each NSD2 siRNA. (**k**) SA-β-Gal staining on day 9 in Ctr- and NSD2-KD Tig-3 cells (each n>300 cells). Data are shown as means ± s.d.; n=3. (**l**) EdU incorporation assay on day 4 of siRNA treatment of top-ranked genes in the screen in IMR-90 cells (each n>240 cells). Data are shown as means ± s.d.; n=3. (**m**) SA-β-Gal staining on day 8 of siRNA treatment of top-ranked genes in the screen in IMR-90 cells (each n>100 cells). Data are shown as means ± s.d.; n=3. (**n**) qRT-PCR of *EZH2* in growing, OIS, early passaged, and late passaged (RS) cells. Data are shown as means ± s.d.; n=3 for OIS and n=1 for RS. (**o**) Fluorescence of NSD2-EGFP on day 6 in growing and OIS IMR-90 ER:Ras cells expressing NSD2-EGFP. Scale bar, 20 μm. (**p**) Western blot analysis of p16 on day 6 in growing and OIS IMR-90 ER:Ras cells expressing NSD2-EGFP. (**q**) SA-β-Gal staining on day 6 in growing and OIS IMR-90 ER:Ras cells expressing NSD2-EGFP (each n>840 cells). Data are shown as means ± s.d.; n=3. Scale bar, 50 μm. *$P < 0.05$, **$P < 0.01$, calculated using Student's *t*-test.

Supporting information Figure 2. Loss of NSD2 alters gene expression in human fibroblasts. Related to Figure 2. (**a**) Enrichment plots of gene set enrichment analysis of the downregulated genes at 24 h in NSD2-KD IMR-90 cells compared with Ctr-KD cells. In each panel, nominal *P*-values and FDR are shown. (**b**) Metascape analysis showing the enriched GO terms in the downregulated or upregulated genes in NSD2-KD IMR-90 cells. (**c**) qRT-PCR of *p21* and *p16* on day 6 in Ctr- and NSD2-KD IMR-90 cells. Data are shown as means ± s.d.; n=3. (**d**) Immunofluorescence of γ-H2AX at 24 h in Ctr- and NSD2-KD IMR-90 cells. Relative fluorescence was calculated by the mean fluorescence of each cell on day 3 (each n>1,300 cells). A topoisomerase I inhibitor (camptothecin, CPT) was used as a positive control. Scale bar, 200 μm. Data are shown as means ± s.d.; n=3. (**e**) qRT-PCR of SASP factors on days 1 and 6 in Ctr- and NSD2-KD IMR-90 cells. SETD8-KD and OIS were used as a positive control. Data are shown as means ± s.d.; n=3. (**f**) Western blot analysis of NSD2 with immunoprecipitated samples using control

IgG or anti-NSD2 antibody. (**g**) Correlation between mRNA expression levels (RNA-seq) and NSD2 enrichment levels (ChIP-seq) at gene bodies of all protein-coding genes. *P < 0.05, **P < 0.01, calculated using Student's *t*-test.

Supporting information Figure 3. Loss of NSD2 does not change the levels of H3K36 mono- and di-methylation at NSD2-enriched gene bodies. Related to Figures 3 and 4. (**a**) ChIP-qPCR of NSD2 at *EZH2* gene locus at 24 h in Ctr- and NSD2-KD IMR-90 cells. Data are shown as means ± s.d.; n=3. (**b**) qRT-PCR of *FN1* and *GAPDH* at 24 h in Ctr- and NSD2-KD IMR-90 cells. *36B4* was used for normalization of *GAPDH*. Data are shown as means ± s.d.; n=3. (**c**) Distribution of NSD2 and modified histone marks around each modification peak region in IMR-90 cells. (**d**) Distribution of NSD2 and modified histone marks around gene loci in IMR-90 cells. (**e**) Immunofluorescence of H3K36me2 at 24 h in Ctr- and NSD2-KD IMR-90 cells. Relative fluorescence was calculated by the mean fluorescence in Ctr- and NSD2-KD cells (each n>600). Data are shown as means ± s.d.; n=3. Scale bar, 100 μm. (**f**) ChIP-qPCR of H3K36me2 using monoclonal antibodies at intergenic (inter), promoter (p), and gene body (b) of indicated gene loci at 24 h in Ctr- and NSD2-KD IMR-90 cells. Primers used are shown in Figure 3b. Data are shown as means ± s.d.; n=3. (**g–i**) ChIP-qPCR of H3K36me2 (using polyclonal and monoclonal antibodies) and H3K36me1 at intergenic (inter), promoter (p), and gene body (b) of indicated gene loci in growing or OIS IMR-90 cells. Primers used are shown in Figure 3b. Values are means ± s.d.; n=3. *P < 0.05, **P < 0.01, calculated using Student's *t*-test.

Supporting information Figure 4. The promoter regions of the downregulated genes in NSD2-KD IMR-90 cells are enriched with RB and RBL2. Related to Figure 5. (**a**) ChIP-Atlas analysis showing top ranked chromatin regulators associated with promoter regions (-0.5 kb < TSS < 0.5 kb) of the NSD2-KD downregulated genes. (**b**) Integrative Genomics Viewer tracks showing RB and RBL2 distribution at the NSD2-KD downregulated gene loci in growing or senescent cells. RB and RBL2 ChIP-seq data in IMR-90 cells were obtained from GSE19899. RB and RBL2 peaks were obtained from the senescent conditions using MACS. (**c**) qRT-PCR of *NSD2* and *RB1* on day 2 in Ctr-, NSD2-, and RB1-KD IMR-90 cells. Data are shown as means ± s.d.; n=3. (**d**) qRT-PCR of *AURKB* and *CCNA2* on day 2 in Ctr-, NSD2-, and RB1-KD IMR-90 cells. Data are shown as means ± s.d.; n=3. (**e**) qRT-PCR of *AURKB* and *CCNA2* on day 2 in Ctr-, NSD2-, and RBL2-KD IMR-90 cells. Data are shown as means ± s.d.; n=3. (**f**) SA-β-Gal staining on day 6 of Ctr-, NSD2-, and RB1-KD IMR-90 cells (each n>55 cells). Data are shown as means ± s.d.; n=3. Scale bar, 200 μm. *P < 0.05, **P < 0.01, calculated using Student's *t*-test.

Supporting information Figure 5. The correlation of gene expression between *NSD2* and genes downregulated in NSD2-KD IMR-90 cells in mammalian cells and tissues. Related to Figure 6. (**a**) Scatter plots showing the correlation between mRNA expression levels of *NSD2* and *MCM3*, *LMNB1*, *EZH2*, *FN1*, and *GAPDH* in 37 human normal tissues. Data were obtained from the HPA. (**b**) Heatmaps showing the correlation between mRNA expression levels of *NSD2* and genes upregulated in NSD2-KD IMR-90 cells in 37 human normal tissues and 1,019 human cancer cell lines. Data were obtained from the HPA and CCLE. Venn diagrams showing the number of negatively correlated genes among genes upregulated in NSD2-KD IMR-90 cells in HPA or CCLE. (**c**) Correlation

between mRNA expression levels of *NSD2* and *p21* in 1,019 human cancer cell lines. Data were obtained from CCLE. (**d**) qRT-PCR of *Nsd2* in 14 normal tissues from 7-week-old male C57BL/6J mice. n=1. (**e**) qRT-PCR of *Nsd2*, *p21*, and *p16* in spleen from 12-week-old and 29–31-month-old female C57BL/6J mice. Data are shown as means ± s.d.; n=3. (**f**) Schematic model of induction of quiescence and serum stimulation combined with NSD2 siRNA treatment in IMR-90 cells. (**g**) qRT-PCR of *FOS* and *JUN* during serum stimulation in Ctr- and NSD2-KD IMR-90 cells. Data are shown as means ± s.d.; n=3. (**h**) Immunofluorescence of NSD2 protein in each cell cycle phase at 25 h in Ctr- and NSD2-KD IMR-90 cells. Each cell cycle phase was defined by the fluorescence intensity of DAPI. Data are shown as means ± s.d.; n=3. Scale bar, 100 μm. (**i**) qRT-PCR of *NSD2* and *MCM3* during serum stimulation in early and late passage IMR-90 cells. *36B4* was used for normalization. Data are shown as means ± s.d.; n=3. *$P < 0.05$, **$P < 0.01$, calculated using Student's *t*-test.

REFERENCES

Subramanian, A., Tamayo, P., Mootha, V. K., Mukherjee, S., Ebert, B. L., Gillette, M. A., … Mesirov, J. P. (2005). Gene set enrichment analysis: a knowledge-based approach for interpreting genome-wide expression profiles. *Proceedings of the National Academy of Sciences of the United States of America*, *102*(43), 15545–15550. https://doi.org/10.1073/pnas.0506580102

Zhou, Y., Zhou, B., Pache, L., Chang, M., Khodabakhshi, A. H., Tanaseichuk, O., … Chanda, S. K. (2019). Metascape provides a biologist-oriented resource for the analysis of systems-level datasets. *Nature Communications*, *10*(1). https://doi.org/10.1038/s41467-019-09234-6

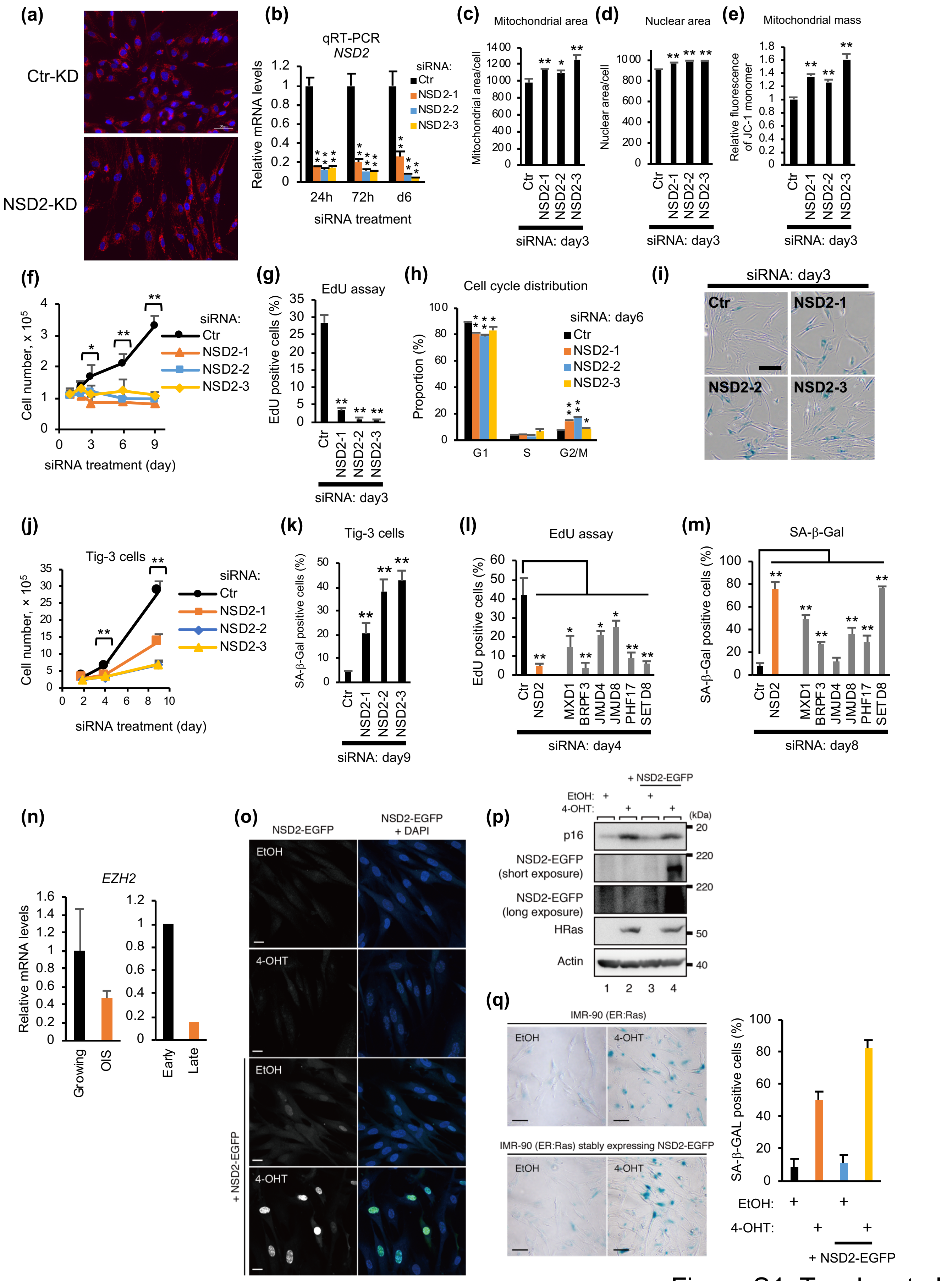

Figure S1 Tanaka et al.

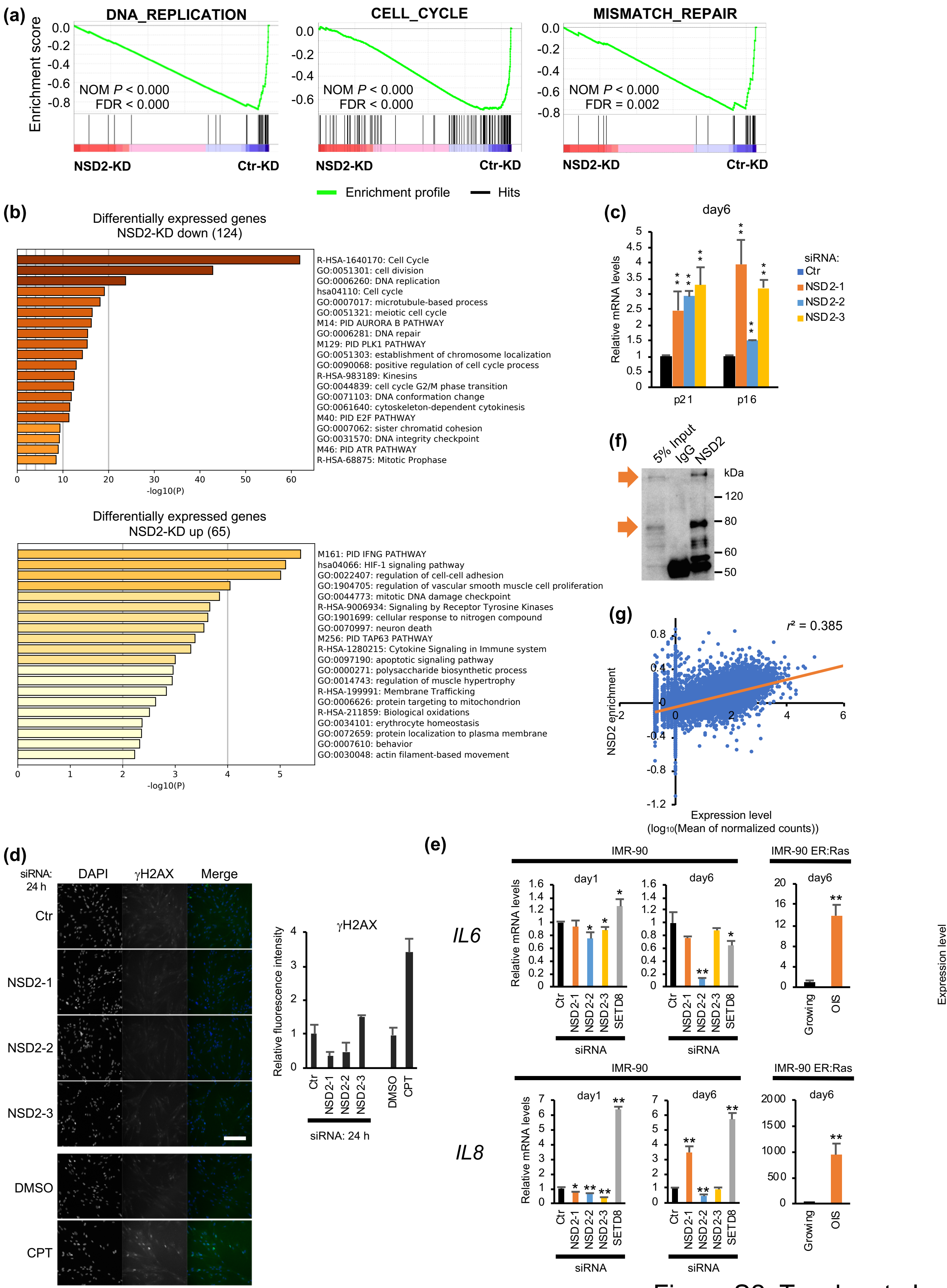

Figure S2 Tanaka et al.

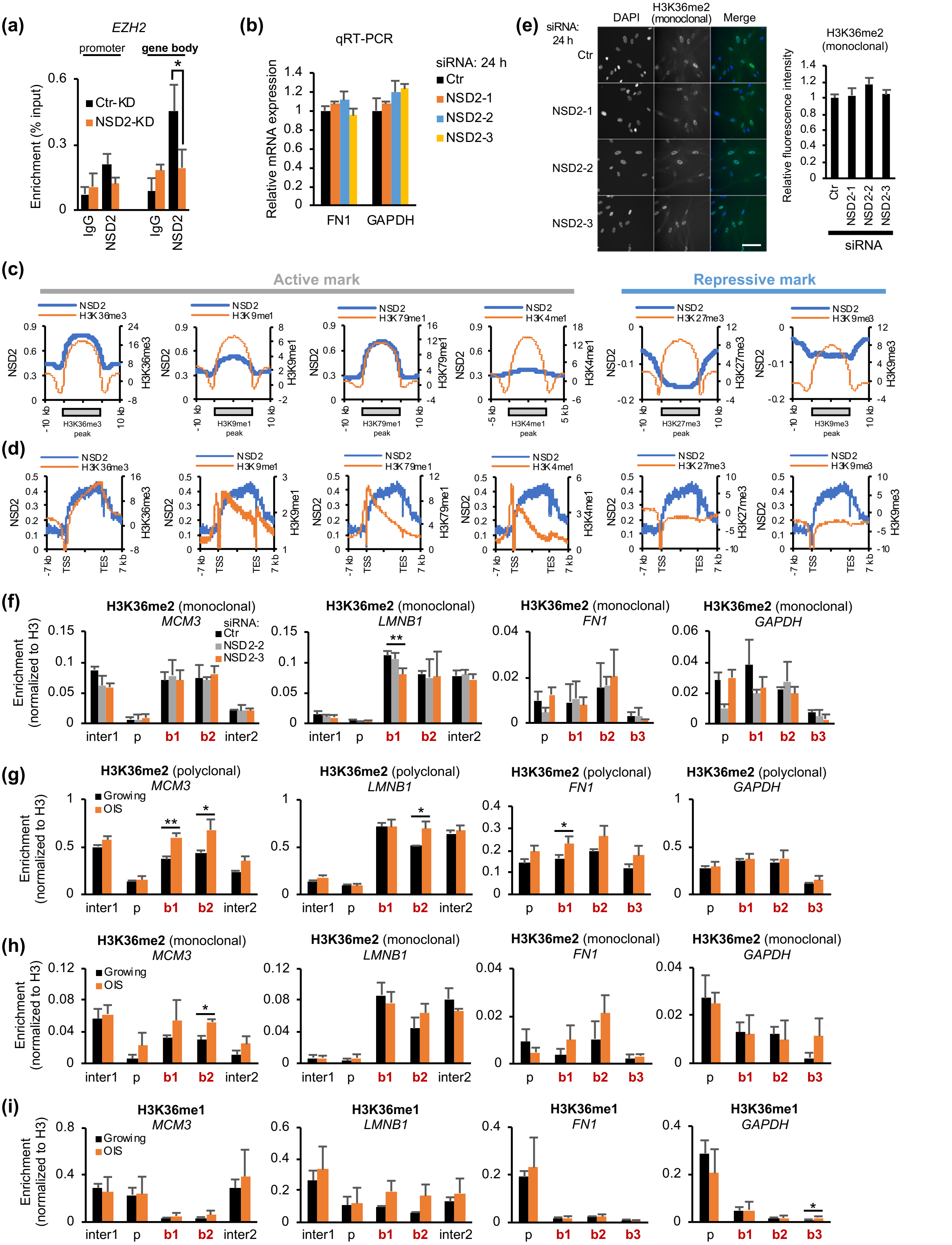

Figure S3 Tanaka et al.

(a) Top ranked promoter-associated chromatin regulators in NSD2-KD downregulated genes

SRA_ID	Antigen	Cell class	Cell	Num. of peaks	Overlaps /My data	Overlaps /Refseq genes	Log P-val	Log Q-val	Fold Enrichment
SRX150410	**E2F4**	Blood	GM12878	2186	111/123	1546/18422	-102.2	-98.3	10.75
SRX027401	**E2F4**	Adipocyte	Adipocytes	1552	107/123	1317/18422	-101.8	-98.2	12.17
SRX016038	**RBL2**	Lung	IMR-90	2240	94/123	736/18422	-101.5	-98.1	19.13
SRX016034	**RBL2**	Lung	IMR-90	3103	92/123	707/18422	-99.4	-96.1	19.49
SRX668217	**E2F4**	Others	HRPEpiC	893	78/123	359/18422	-96.5	-93.3	32.54
SRX016031	**RBL2**	Lung	IMR-90	2584	85/123	598/18422	-92.8	-89.7	21.29
SRX194567	**E2F4**	Breast	MCF-7	947	84/123	601/18422	-90.9	-87.8	20.93
SRX194566	**E2F4**	Breast	MCF-7	2398	106/123	1714/18422	-88.3	-85.3	9.26
SRX150445	**E2F4**	Uterus	HeLa	2645	111/123	2186/18422	-86.2	-83.3	7.61
SRX019963	**E2F4**	Blood	GM06990	359	70/123	309/18422	-86.2	-83.3	33.93
SRX1548208	**E2F1**	Cardiovascular	Wharton Jelly	664	73/123	463/18422	-80.1	-77.3	23.61
SRX396584	**RNF2**	Epidermis	Hs 68	2074	77/123	587/18422	-79.7	-76.9	19.65
SRX1497386	**E2F1**	Blood	RAJI	1580	88/123	1202/18422	-73.3	-70.7	10.96
SRX1548205	**E2F1**	Cardiovascular	Wharton Jelly	740	66/123	470/18422	-68.2	-65.6	21.03
SRX1548207	**E2F1**	Cardiovascular	Wharton Jelly	1183	77/123	884/18422	-67.1	-64.5	13.05

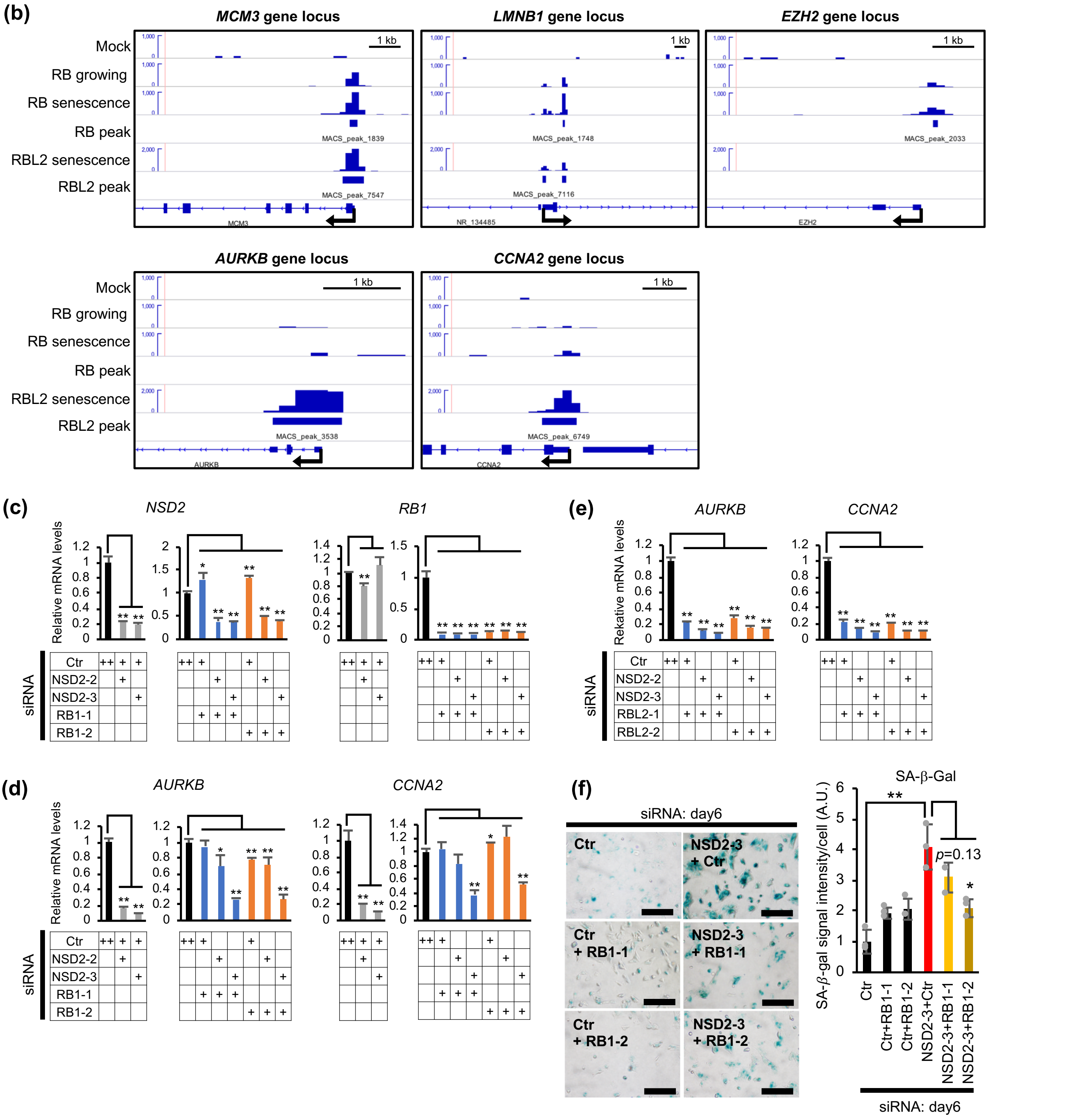

Figure S4 Tanaka et al.

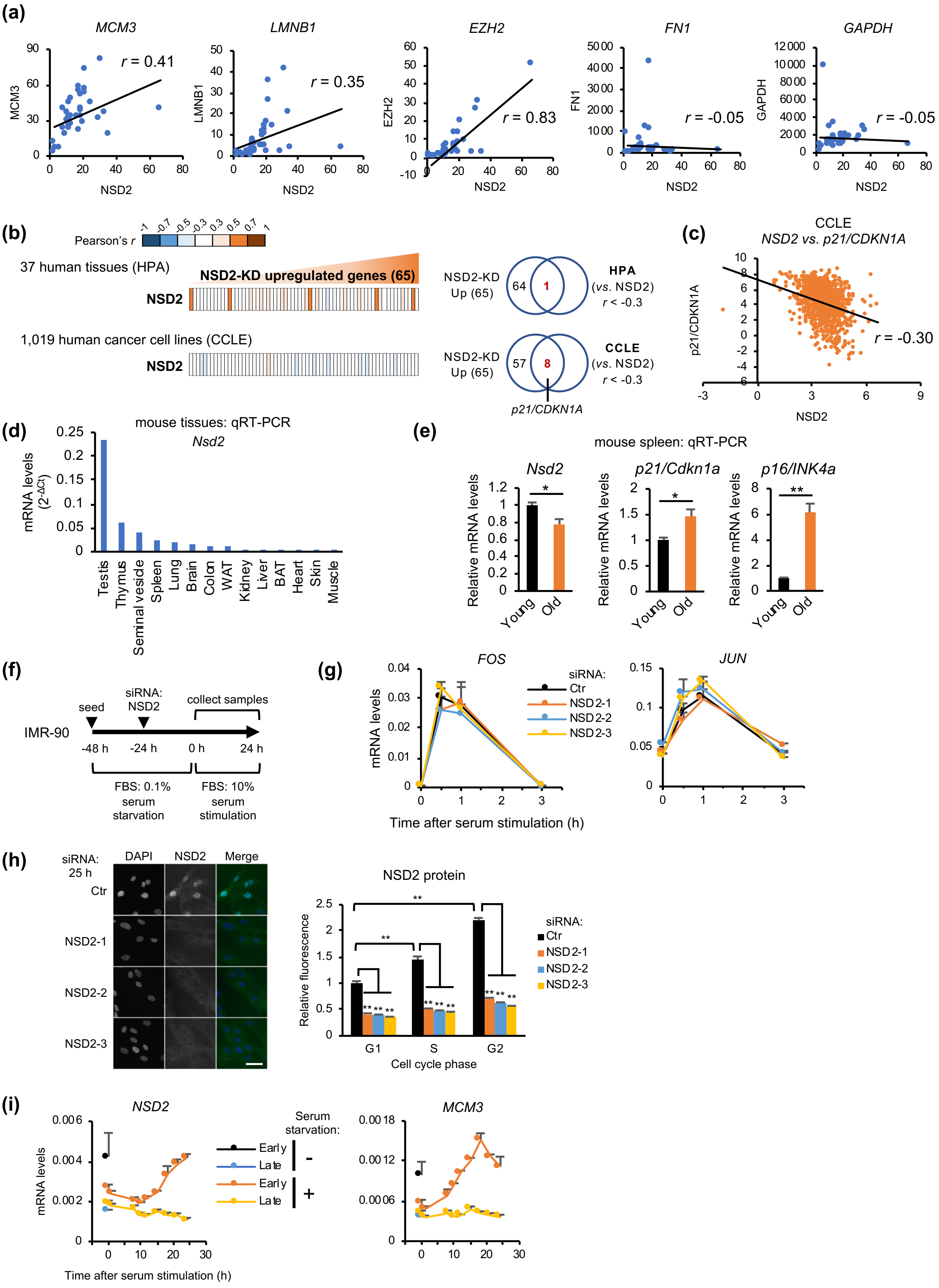

Figure S5 Tanaka et al.

SUPPORTING INFORMATION TABLE S1. Primers used in this study. Related to Figures 1, 2, 5, 6, and Supporting information Figures S1–S5.

**qRT-PCR primers**		
**Target**	**Forward primer**	**Reverse primer**
**Human**		
NSD2	5′-GTTTCTGCAGATCCACTCCTTC-3′	5′-GGCTCTTCTCAAATATCCAAGC-3′
p16/INK4A	5′-CAACGCACCGAATAGTTACG-3′	5′-CTGCCCATCATCATGACCT-3′
p21/CDKN1A	5′-CATGGGTTCTGACGGACATC-3′	5′-TGCCGAAGTCAGTTCCTTGT-3′
MCM3	5′-GGGTGGAACGAGACCTAGAA-3′	5′-AGACTTGGCAACGGATGG-3′
CCNA2	5′-GGTACTGAAGTCCGGGAACC-3′	5′-GAAGATCCTTAAGGGGTGCAA-3′
LMNB1	5′-AAGCCGCATGAGAATTGAGAG-3′	5′-CTTGAATCCTTTCCAAACATGC-3′
AURKB	5′-TCTCTAAGGATGGCCCAGAAG-3′	5′-CATGAGGACAAGTGCAGATGG-3′
BARD1	5′-TTGAAGTGTATGCTTGGGATTC-3′	5′-TCCTGTTCACATACTTTTCTTCG-3′
BRCA1	5′-CTGAAGACTGCTCAGGGCTATC-3′	5′-CATTTCCTGCTGGAGCTTTATC-3′
RAD51	5′-AGGGTACCTTTAGGCCAGAAC-3′	5′-TACATTATCCAGGACATCACTGC-3′
EZH2	5′-GGGACAGTAAAAATGTGTCCTGC-3′	5′-TGCCAGCAATAGATGCTTTTTG-3′
ATAD2	5′-TTCCTCAGTCTGGAGCACATCG-3′	5′-CCGGTGGTAGGTTTCAACTTCC-3′
UHRF1	5′-AAGATCCAGGAGCTGTTCCAC-3′	5′-ACCTCGTAGTCGAAGAGGGTATG-3′
IL6	5′-CCAGGAGCCCAGCTATGAAC-3′	5′-CCCAGGGAGAAGGCAACTG-3′
IL8	5′-AAGGAAAACTGGGTGCAGAG-3′	5′-ATTGCATCTGGCAACCCTAC-3′
CCL2	5′-ATAGCAGCCACCTTCATTCC-3′	5′-GCACTGAGATCTTCCTATTGGTG-3′
CXCL1	5′-CCTGCATCCCCCATAGTTAAG-3′	5′-AGTGAGCTTCCTCCTCCCTTC-3′
FN1	5′-GAGGAGAGTGGAAGTGTGAGAG-3′	5′-GTAAACAGCTGCACGAACATC-3′
RB1	5′-TCAGTTGGTCCTTCTCGGTC-3′	5′-TGTGAACATCGAATCATGGAA-3′
RBL2	5′-AGGAGTTTCTCCTGTGCGTATC -3′	5′-TGAAACAATGCTTTCTCCTCG -3′
FOS	5′-CTTACTACCACTCACCCGCA-3′	5′-GGGAATGAAGTTGGCACTGG-3′
JUN	5′-CCTCAACGCCTCGTTCCT-3′	5′-AGTTCTTGGCGCGGAGGT-3′
NSD1	5′-TTACCTGGAACTTCATCATCATC-3′	5′-CTTCATACTTCAGTGGCGTAGAC-3′
NSD3	5′-ATTGTTCTGCTCCAGCATCC-3′	5′-TCTCTCCTGTGATCGCACTG-3′
SETD2	5′-GGAAAATGAAGAAGGAACGATCTC-3′	5′-CACCATTTTCCATCAGAGCTTC-3′
SETD3	5′-AAACACAGCTCGACAGTACGC-3′	5′-ACCTGTAGTCCTCGTAAGTGAAA-3′
SETMAR	5′-GTTGTGGAAATGAGAAGGAACC-3′	5′-GACCCATTTTGAACTCGAATAAG-3′
ASH1L	5′-ACTTCCCTGCACTCTCATCG-3′	5′-AAACAAGGATCTCCAAAACTCG-3′
SMYD2	5′-AACCACTGCGAGTACTGCTTC-3′	5′-CTTCTTTCTGACACTCCACATTG-3′
GAPDH (control)	5′-ACACCCACTCCTCCACCTTT-3′	5′-TAGCCAAATTCGTTGTCATACC-3′
ACTB (control for GAPDH)	5′-CCAACCGCGAGAAGATGA-3′	5′-CCAGAGGCGTACAGGGATAG-3′
36B4 (control for quiescent cells)	5′-GATGCCCAGGGAAGACAG-3′	5′-TCTGCTCCCACAATGAAACAT-3′
**Mouse**		
Nsd2	5′-CCCACTCCTTCACAATCATACC-3′	5′-TCAAATATCCAAGCTCTTTCTGG-3′
p16/INK4a	5′-CCCAACGCCCCGAACT-3′	5′-GCAGAAGAGCTGCTACGTGAA-3′
Mcm3	5′-GCAGAGAGACTACTTGGACTTCC-3′	5′-TGTCACTGATCAGTTCCCGAAC-3′
Lmnb1	5′-ATCGAGCTGGGCAAGTTCAAG-3′	5′-GGCTCCACTGAGATCAGATTCC-3′
Aurkb	5′-TTCGAGAGCCCTAGCCACAG-3′	5′-GAAGGCACAGAAGAGGGGAAC-3′
Fn1	5′-CGACATGCTCTACAAAGTGCTTC-3′	5′-GCGGTTGGTAAATAGCTGTTCG-3′
Gapdh (control)	5′-GTGGACCTCATGGCCTACAT-3′	5′-GGGTGCAGCGAACTTTATTG-3′
36b4 (control for Gapdh)	5′-GCGTCCTGGCATTGTCTGT-3′	5′-GCAAATGCAGATGGATCAGCC-3′

SUPPORTING INFORMATION TABLE S1. Primers used in this study. Related to Figures 1, 2, 5, 6, and Supporting information Figures S1–S5.

**ChIP-qPCR primers**		
**Target**	**Forward primer**	**Reverse primer**
MCM3-inter1	5′-GTCCCTTCCAAATTTGTGATCC-3′	5′-ACACACCCCTACTTCCAAGACTG-3′
MCM3-p	5′-TTCTGGGAGTTGTAGTGTTCTCC-3′	5′-GGTCAAAAGTTCTGCAGCTTG-3′
MCM3-b1	5′-TTATGTTTCATTTCTCCACCACTG-3′	5′-AAGCACTATTAGGCGCTATGGAG-3′
MCM3-b2	5′-AAGAGTTCTGGACTTGGGTCTTTC-3′	5′-TGTCTAAATCTTTTAGGGGCAGTG-3′
MCM3-inter2	5′-ATAAGGGTTGTTGTAGGGAAGTG-3′	5′-GCTTGCTTCACTAATTGGTCTC-3′
LMNB1-inter1	5′-TATTTTGACAGCACAAAAGTGTTC-3′	5′-CCAAAATTATTTAGCCAGTTATGC-3′
LMNB1-p	5′-AAGACGCACAGATCTCACTTTC-3′	5′-GTACCAGCCTTTATTTTTCTCTCC-3′
LMNB1-b1	5′-TTCATTTGCTAAAGAAAGGTGAAG-3′	5′-CATGGAATAAAGTTGGAAAGTAGC-3′
LMNB1-b2	5′-AAACAACCATACCTGAAGAAGAGG-3′	5′-AATACCTGCTGGTGGAAAAGTTC-3′
LMNB1-inter2	5′-TATGATGTGAGTGCTTCCTCCAC-3′	5′-GCATTTCTCTAAAAGCAAACTTGG-3′
FN1-p	5′-CTGGACTTGTGTGAAGCGAAG-3′	5′-CGCAATGTCCTCAAACACTACC-3′
FN1-b1	5′-TGCCACTCCATACAGCAGATAC-3′	5′-CTCCAGAAAATCCAGGACAGC-3′
FN1-b2	5′-CTCAAGCCAAAGTGGAGTACAAC-3′	5′-GTGGAATTTCACACCAGTGCTC-3′
FN1-b3	5′-ACAGCATGGAAGCAGCAATAC-3′	5′-AGTATGAGAAGCCTGGGTCTCC-3′
GAPDH-p	5′-CAATTCCCCATCTCAGTCGTTCC-3′	5′-AGCAGGACACTAGGGAGTCAAG-3′
GAPDH-b1	5′-TTTATGGAGGTCCTCTTGTGTCC-3′	5′-TTCCAACTACCCATGACTCAGC-3′
GAPDH-b2	5′-GCTTGCCCTGTCCAGTTAATTTC-3′	5′-CGCTTGTACACTCAGCATCATC-3′
GAPDH-b3	5′-CAACGACCACTTTGTCAAGCTC-3′	5′-GCCAGACCCTGCACTTTTTAAG-3′
EZH2-p	5′-ATTATGTCTGCTGCTGCCTTG-3′	5′-CACAGGTTTCTAGGGCGATAAG-3′
EZH2-gene body	5′-AAAACTGAAGAGACTGCCCAAG-3′	5′-AGGTGCAACTGATGAGAAAGTG-3′

SUPPORTING INFORMATION TABLE S2. siRNAs used in this study.

**siRNAs**		
**Target**		
siNSD2-1 (targeted to coding region)	5′-GAAAAUCACCAAAACAUAC-3′	
siNSD2-2 (targeted to coding region)	5′-CAAACUUAAAGGUCAGAAA-3′	
siNSD2-3 (targeted to coding region)	5′-AGAAGACUCUGGUAAUGUA-3′	
siRB1-1	sc-44273B	Santa Cruz Biotechnology
siRB1-2	sc-44273C	
siRBL2-1	5′-GGUAAUUUCCCCAUGAUUA-3′	
siRBL2-2	5′-CUAUAUUAGGAAACUUUAU-3′	
siCtr (targeted to luciferase GL3)	5′-CUUACGCUGAGUACUUCGA-3′	

SUPPORTING INFORMATION TABLE S2. siRNAs used in this study.

Gene Symbol	RefSeq Accession Number	siRNA ID A	siRNA ID B	siRNA ID C	
RERE	NM_001042681	s1713	s1712	s1711	Silencer Select (Thermo Fisher Scientific)
HOXA6	NM_024014	s223863	s6767	s6768	
ZNF48	NM_152652	s47108	s47106	s47107	
KIAA1310	NM_017991	s31243	s31244	s31242	
SMYD2	XM_001127274	s32469	s32468	s32470	
TDRKH	NM_001083963	s21712	s21710	s21711	
DNAJA2	NM_005880	s20123	s20122	s20124	
MBD3	NM_003926	s28738	s28737	s28736	
CHD8	NM_020920	s33580	s33582	s33581	
SETD8	NM_020382	s51990	s51988	s51989	
MBD3L1	NM_145208	s40081	s40082	s40083	
ZNF521	NM_015461	s24760	s24762	s24761	
IKZF5	NM_022466	s34665	s34667	s34666	
ZNF467	NM_207336	s46720	s46718	s46719	
TRIP12	NM_004238	s17810	s17808	s17809	
ZNF454	NM_182594	s50007	s50008	s50009	
FEZF2	NM_018008	s30121	s30120	s30122	
MSS51	NM_001024593	s42222	s4221	s223249	
C5orf35	NM_153706	s43769	s43770	s43771	
ZHX3	NM_015035	s22922	s22921	s22920	
GFI1B	NM_004188	s15851	s15852	s15850	
SOX4	NM_003107	s224666	s13300	s13301	
SMARCD2	NM_003077	s13156	s13155	s13154	
UBE2A	NM_003336	s14566	s14567	s14565	
C14orf169	NM_024644	s36124	s36125	s36123	
BPTF	XM_929003	s197300	s197301	s5009	
JMJD4	NM_023007	s195361	s35205	s35204	
PAX9	NM_006194	s10076	s10077	s10078	
BRD1	NM_014577	s24388	s24389	s24390	
NFXL1	NM_152995	s45701	s45700	s45702	
WHSC1	NM_007331	s200462	s200461	s200460	
WHSC1L1	NM_017778	s29726	s29727	s29725	
TSHZ3	NM_020856	s33448	s33450	s33449	
INO80C	NM_194281	s42888	s42886	s42887	
ELP4	NM_019040	s25575	s25577	s25576	
SUPV3L1	NM_003171	s13637	s13639	s13638	
SMAD1	NM_001003688	s8395	s8394	s8396	
SMARCD1	NM_003076	s13151	s13152	s13153	
CTDSP1	NM_021198	s33805	s33804	s33806	
JMJD8	NM_001005920	s50457	s50456	s50455	
ASXL2	NM_018263	s30558	s30557	s30559	
DNLZ	NM_001080849	s58680	s227627	s58681	
PHF8	NM_015107	s23108	s23107	s23106	
C12orf41	NM_017822	s29793	s29791	s29792	
PRDM1	NM_182907	s1993	s1992	s1991	
ZNF395	NM_018660	s31734	s31735	s31733	
ZCCHC4	XM_940889	s26408	s26409	s26410	
ZNF607	NM_032689	s39423	s39425	s39424	
LIMK2	NM_001031801	s8191	s8192	s8193	
AICDA	NM_020661	s32974	s32975	s224465	
ZMYM2	NM_003453	s15268	s15266	s15267	
MBD3L2	XM_001130308	s59756	s199174	s199173	
SETD3	NM_032233	s38640	s38641	s38639	
NFATC4	NM_004554	s9484	s9482	s9483	
ZNF677	NM_182609	s50952	s50951	s50950	
RNF112	NM_007148	s15239	s15241	s15240	
MNF1	NM_032340	s38860	s38859	s38858	
FOXF1	XM_001128652	s226532	s53295	s226163	
BRPF3	NM_015695	s25920	s25919	s25918	
KDM6B	NM_001080424	s23109	s23110	s23111	
MORC1	NM_014429	s25888	s25890	s25889	
PHF17	NM_024900	s36726	s36724	s36725	
OTP	NM_032109	s223596	s195104	s223597	
GPN1	NM_007266	s22322	s22323	s22324	
MTERFD1	NM_015942	s27209	s27210	s27211	
BAHD1	NM_014952	s22604	s22606	s22605	
HOXC11	NM_014212	s6833	s6835	s6834	
ZNF26	NM_019591	s15062	s15060	s15061	
TDRD5	NM_173533	s46464	s46463	s46462	
MXD1	NM_002357	s8388	s8390	s8389	
SETD4	NM_001007259	s28859	s28857	s28858	
JHDM1D	NM_030647	s37450	s37449	s37448	
ZNF687	NM_020832	s33395	s33396	s33394	
NFKB1	NM_003998	9505	9506	9504	
HDAC6	NM_006044	19460	19459	19461	
HDAC7	NM_015401	28336	28335	28337	
HDAC8	NM_018486	31697	31698	31699	
HDAC11	NM_024827	36568	36567	36566	
ZMAT2					Dharmacon smartpool (Thermo Fisher Scientific)
