# Supplementary material for: The NSD2/WHSC1/MMSET methyltransferase prevents cellular senescence‐associated epigenomic remodeling
Source: Aging Cell. 2020 Jun 22;19(7):e13173. doi: 10.1111/acel.13173 (PMC7433007; doi:10.1111/acel.13173)
Supplement: Supplementary file 2 — Table S3 [file ACEL-19-e13173-s002.pdf]

**: p <0.01
*: p < 0.05

plate1-1	Mito area	plate1-2	Mito area	plate1-3	Mito area		MEAN	SD	TTEST	Significance
siGL3	640.34	siGL3	643.48	siGL3	649.42	siGL3	644.413333	4.61139169		
RERE	852.95	RERE	879.11	RERE	878.06	RERE	870.04	14.8096826	1.4735E-05	**
HOXA6	524.37	HOXA6	535.84	HOXA6	585.01	HOXA6	548.406667	32.2140223	0.00693465	**
ZNF48	946.01	ZNF48	803.45	ZNF48	803.52	ZNF48	850.993333	82.2868546	0.01223872	*
KIAA1310/K/	778.08	KIAA1310/K/	636.54	KIAA1310/K/	745.28	KIAA1310/K/	719.966667	74.0875599	0.15269795	
SMYD2	632.95	SMYD2	664.34	SMYD2	807.95	SMYD2	701.746667	93.3043088	0.34769483	
TDRKH	629.23	TDRKH	655.08	TDRKH	748	TDRKH	677.436667	62.4615372	0.41277841	
DNAJA2	649.09	DNAJA2	716.81	DNAJA2	695.23	DNAJA2	687.043333	34.594302	0.10183817	
ZNF521	837.38	ZNF521	825.66	ZNF521	824.25	ZNF521	829.096667	7.20813661	3.0578E-06	**
IKZF5	808.67	IKZF5	686.69	IKZF5	750.01	IKZF5	748.456667	61.0048337	0.04215356	*
ZNF467	803.07	ZNF467	714.79	ZNF467	672.68	ZNF467	730.18	66.5434227	0.0899031	
TRIP12	829.27	TRIP12	643.09	TRIP12	806.23	TRIP12	759.53	101.49589	0.12121118	
ZNF454	903.16	ZNF454	804.88	ZNF454	893.97	ZNF454	867.336667	54.2838874	0.00209251	**
FEZF2	776.05	FEZF2	708.01	FEZF2	808.17	FEZF2	764.076667	51.1422226	0.01565175	*
MSS51	874.35	MSS51	898.09	MSS51	834.85	MSS51	869.096667	31.9456184	0.00027135	**
siGL3	776.08	siGL3	692.37	siGL3	657.69	siGL3	708.713333	60.8635887		
SMARCD2	544.32	SMARCD2	585.91	SMARCD2	520.99	SMARCD2	550.406667	32.885213	0.01662846	*
UBE2A	570.87	UBE2A	444.08	UBE2A	550.17	UBE2A	521.706667	68.0187256	0.02382606	*
C14orf169/N	761.33	C14orf169/N	668.77	C14orf169/N	792.11	C14orf169/N	740.736667	64.1969854	0.56466853	
BPTF	768.16	BPTF	829.08	BPTF	788.41	BPTF	795.216667	31.0251452	0.09335842	
JMJD4	998.92	JMJD4	914.49	JMJD4	840.18	JMJD4	917.863333	79.423746	0.02235084	*
PAX9	871.29	PAX9	765.53	PAX9	753.3	PAX9	796.706667	64.879877	0.16182534	
BRD1	790.2	BRD1	729.44	BRD1	728.84	BRD1	749.493333	35.2542839	0.37209556	
INO80C	743.68	INO80C	665.95	INO80C	729.67	INO80C	713.1	41.4296017	0.92277403	
ELP4	669.81	ELP4	595.39	ELP4	607.19	ELP4	624.13	39.9975849	0.11459297	
SUPV3L1	865.3	SUPV3L1	864.36	SUPV3L1	754.45	SUPV3L1	828.036667	63.7296558	0.07892279	
SMAD1	841.16	SMAD1	788.42	SMAD1	743.11	SMAD1	790.896667	49.0718966	0.14276157	
SMARCD1	690.94	SMARCD1	667.67	SMARCD1	681.22	SMARCD1	679.943333	11.6874135	0.46643789	
CTDSP1	783.18	CTDSP1	732.24	CTDSP1	667.74	CTDSP1	727.72	57.8525816	0.71501894	
JMJD8	988.81	JMJD8	916.27	JMJD8	806.32	JMJD8	903.8	91.8818573	0.03744183	*
siGL3	690.93	siGL3	624.04	siGL3	658.07	siGL3	657.68	33.4467054		
MBD3	771.92	MBD3	592.99	MBD3	725.18	MBD3	696.696667	92.8033482	0.5309423	
CHD8	601.13	CHD8	539.22	CHD8	640.9	CHD8	593.75	51.2401591	0.14461373	
SETD8	944.63	SETD8	797.85	SETD8	899.26	SETD8	880.58	75.151839	0.00935397	**
MBD3L1	726.53	MBD3L1	721.87	MBD3L1	786.64	MBD3L1	745.013333	36.1249697	0.03719974	*
PRDM1	579.49	PRDM1	582.87	PRDM1	712.01	PRDM1	624.79	75.5536392	0.52844658	
ZNF395	829.04	ZNF395	694.31	ZNF395	843.46	ZNF395	788.936667	82.2656589	0.0626362	
ZCCHC4	887.98	ZCCHC4	752.39	ZCCHC4	900.15	ZCCHC4	846.84	82.0221257	0.0208579	*
C5orf35/SET	942.72	C5orf35/SET	694.21	C5orf35/SET	919.74	C5orf35/SET	852.223333	137.32509	0.07565625	
ZHX3	736.15	ZHX3	674	ZHX3	700.85	ZHX3	703.666667	31.1705924	0.15644154	
GFI1B	750.2	GFI1B	648.53	GFI1B	745.25	GFI1B	714.66	57.323715	0.21120759	
SOX4	887.13	SOX4	774.06	SOX4	830.35	SOX4	830.513333	56.535177	0.01036047	*
RNF112	765.94	RNF112	662.88	RNF112	738.81	RNF112	722.543333	53.4209157	0.14924907	
MNF1	872.91	MNF1	740.58	MNF1	806.35	MNF1	806.613333	66.165393	0.02536518	*
FOXF1	857.72	FOXF1	841.28	FOXF1	846.17	FOXF1	848.39	8.44184222	0.00066456	**

plate2-1	Mito area	plate2-2	Mito area	plate2-3	Mito area		MEAN	SD	TTEST	
siGL3	703.93	siGL3	667.15	siGL3	755.52	siGL3	708.866667	44.3913531		
NFXL1	748.93	NFXL1	733.27	NFXL1	793	NFXL1	758.4	30.9706135	0.18813562	
NSD2/WHSC	1074.34	NSD2/WHSC	947.97	NSD2/WHSC	975.03	NSD2/WHSC	999.113333	66.5383306	0.00327312	**
WHSC1L1	719.33	WHSC1L1	652.81	WHSC1L1	794.77	WHSC1L1	722.303333	71.0266917	0.7948902	
TSHZ3	720.03	TSHZ3	635.16	TSHZ3	704.79	TSHZ3	686.66	45.2465678	0.57673767	
HOXC11	799.04	HOXC11	819.94	HOXC11	859.74	HOXC11	826.24	30.8365043	0.01975404	*
ZNF26	754.49	ZNF26	725.91	ZNF26	870.66	ZNF26	783.686667	76.6646896	0.21733848	
TDRD5	728.86	TDRD5	706.51	TDRD5	751.27	TDRD5	728.88	22.3800067	0.52403325	
ASXL2	653.45	ASXL2	641.35	ASXL2	670.86	ASXL2	655.22	14.8344093	0.11808934	
DNLZ	715.85	DNLZ	724.71	DNLZ	816.89	DNLZ	752.483333	55.9534533	0.34983115	
PHF8	920.28	PHF8	675.33	PHF8	918.58	PHF8	838.063333	140.933764	0.20447848	
C12orf41/KA	804.58	C12orf41/KA	692.49	C12orf41/KA	891.06	C12orf41/KA	796.043333	99.5598676	0.23824157	
siGL3	733.5	siGL3	695.08	siGL3	674.42	siGL3	701	29.981601		
ZNF607	772.07	ZNF607	797.16	ZNF607	819.48	ZNF607	796.236667	23.7184829	0.01249757	*
LIMK2	745.79	LIMK2	683.86	LIMK2	734.37	LIMK2	721.34	32.9570463	0.47334722	
AICDA	782.75	AICDA	754.43	AICDA	786.79	AICDA	774.656667	17.6328935	0.0214314	*
ZMYM2	836.05	ZMYM2	711.92	ZMYM2	690.28	ZMYM2	746.083333	78.6611291	0.40611838	
MBD3L2	758.36	MBD3L2	776.27	MBD3L2	738.17	MBD3L2	757.6	19.0613667	0.05088332	
SETD3	779.07	SETD3	565.04	SETD3	695.95	SETD3	680.02	107.900574	0.76183214	
NFATC4	674.5	NFATC4	642.74	NFATC4	620.07	NFATC4	645.77	27.3412125	0.07786986	
BRPF3	1042.57	BRPF3	955.13	BRPF3	922.65	BRPF3	973.45	62.023531	0.00237721	**
KDM6B	966.5	KDM6B	782.77	KDM6B	820.93	KDM6B	856.733333	96.9566255	0.05651547	
MORC1	874.85	MORC1	816.21	MORC1	733.49	MORC1	808.183333	71.0210035	0.07369973	
PHF17	965.7	PHF17	876.78	PHF17	824.2	PHF17	888.893333	71.5235076	0.01373607	*
OTP	844.43	OTP	746.57	OTP	720.6	OTP	770.533333	65.3004459	0.16902601	
GPN1	837.89	GPN1	777.89	GPN1	745.9	GPN1	787.226667	46.7003216	0.05459222	
MTERFD1	683.47	MTERFD1	637.2	MTERFD1	629.68	MTERFD1	650.116667	29.1285295	0.10268058	
siGL3	783.01	siGL3	701.31	siGL3	758.64	siGL3	747.653333	41.9434457		
MXD1	1035.31	MXD1	993.92	MXD1	1014.12	MXD1	1014.45	20.6969732	0.00058889	**
SETD4	826.95	SETD4	711.95	SETD4	856.77	SETD4	798.556667	76.4711981	0.36925748	
JHDM1D	793.79	JHDM1D	680.67	JHDM1D	743.71	JHDM1D	739.39	56.683599	0.84906295	
ZNF687	934.52	ZNF687	802.74	ZNF687	885.91	ZNF687	874.39	66.6410151	0.04942434	*
ZNF677	875.88	ZNF677	685.72	ZNF677	869.96	ZNF677	810.52	108.120496	0.4009284	
BAHD1	704.24	BAHD1	601.22	BAHD1	703.58	BAHD1	669.68	59.2890175	0.13646431	
ZMAT2	702.63	ZMAT2	657.98	ZMAT2	785.4	ZMAT2	715.336667	64.6533729	0.50787339	
NFKB1	724.48	NFKB1	688.25	NFKB1	827.36	NFKB1	746.696667	72.1670509	0.98511278	
HDAC6	791.62	HDAC6	677.15	HDAC6	725.67	HDAC6	731.48	57.4557421	0.71382454	
HDAC7	679.57	HDAC7	623.12	HDAC7	734.81	HDAC7	679.166667	55.8460924	0.16466035	
HDAC8	773.63	HDAC8	642.79	HDAC8	756.99	HDAC8	724.47	71.2245688	0.65252096	
HDAC11	766.22	HDAC11	731.2	HDAC11	842.34	HDAC11	779.92	56.8224639	0.47303837	

Supporting information Table S3
